# Supplementary material for: Economic viability requires higher recycling rates for imported plastic waste than expected
Source: Nat Commun. 2024 Aug 31;15:7578. doi: 10.1038/s41467-024-51923-4 (PMC11365935; doi:10.1038/s41467-024-51923-4)
Supplement: Supplementary file 1 — Supplementary Information [file 41467_2024_51923_MOESM1_ESM.pdf]

Supplementary information

Economic viability requires higher recycling rates for imported plastic waste than expected

Kai Li [1]*, Hauke Ward [1], Hai Xiang Lin [1,2], and Arnold Tukker [1,3]

[1] Institute of Environmental Sciences (CML), Leiden University, 2333 CC, Leiden, The Netherlands

[2] Delft Institute of Applied Mathematics, Delft University of Technology, 2628 CD, Delft, The Netherlands

[3] Netherlands Organization for Applied Scientific Research TNO, 2595 DA, The Hague, The Netherlands

* Corresponding author: Kai Li, k.li@cml.leidenuniv.nl

This document includes:

Supplementary Tables 1-15, pages 2–28

Supplementary Figures 1-15, pages 29–43

Supplementary References, pages 44–57

1. Supplementary Tables

Supplementary Table 1: Comparison of the required recycling rate using trade data reported by 22 research countries and their trading partners (mirror trade data). The trade data from the 22 research countries include plastic waste imports and primary plastics exports, while the mirror trade data include plastic waste exports and primary plastics imports reported by the trading partners of the 22 research countries. The required recycling rate for each country was averaged over the period 2013–2022.

Country	PE		PS		PVC		Others	
	22 countries	Trading partners	22 countries	Trading partners	22 countries	Trading partners	22 countries	Trading partners
Belgium	0.709	0.696	0.537	0.630	0.883	0.820	0.763	0.748
Canada	0.642	0.680	0.774	0.777	0.808	0.841	0.778	0.763
China	0.580	0.563	0.681	0.571	0.672	0.640	0.725	0.606
Hong Kong (China)	0.518	0.614	0.424	0.448	0.518	0.526	0.515	0.573
Czech Republic	0.625	0.599	0.484	0.502	0.507	0.566	0.513	0.482
France	0.748	0.842	0.482	0.532	0.844	0.851	0.822	0.812
Germany	0.663	0.665	0.647	0.655	0.829	0.852	0.658	0.704
India	0.572	0.532	0.503	0.439	0.572	0.627	0.644	0.583
Indonesia	0.459	0.447	0.409	0.468	0.537	0.509	0.668	0.549
Italy	0.763	0.786	0.709	0.685	0.630	0.696	0.818	0.835
Malaysia	0.657	0.593	0.592	0.491	0.609	0.584	0.650	0.656
Mexico	0.466	0.557	0.507	0.514	0.563	0.665	0.660	0.610
Netherlands	0.618	0.710	0.676	0.712	0.833	0.856	0.781	0.748
Taiwan (China)	0.568	0.546	0.578	0.472	0.614	0.584	0.756	0.631
Republic of Korea	0.642	0.572	0.701	0.612	0.748	0.680	0.704	0.725
Slovenia	0.472	0.474	0.510	0.386	0.773	0.651	0.536	0.542
Spain	0.689	0.690	0.549	0.505	0.815	0.847	0.816	0.780
Thailand	0.432	0.410	0.461	0.404	0.665	0.604	0.509	0.509
Turkey	0.435	0.397	0.502	0.446	0.475	0.406	0.485	0.447
USA	0.813	0.695	0.627	0.562	0.885	0.825	0.744	0.715
UK	0.852	0.800	0.586	0.655	0.846	0.890	0.885	0.894
Vietnam	0.535	0.480	0.506	0.503	0.625	0.460	0.637	0.528

Supplementary Table 2: Mechanical recycling costs of plastic waste from literature review.

Reference	Country	Data year	Plastic waste type	Recycling cost (per kg)
Gradus et al., 2017 [1]	Netherlands	2017-2019	-	0.269 Euro
Lase et al., 2023[2]	Belgium	2018	PE	0.545 Euro
Larrain et al., 2021[3]	European countries	2019	PP, PS, and PE	0.363 Euro (PP)<br>0.348 Euro (PS)<br>0.424 Euro (PE)
Kim et al., 2023[4]	Korea	2020	PVC	0.180-0.210 USD
Nikiema and Asiedu, 2022[5]	-	literature review (2012-2021)	PP, PET, PVC	0.003-0.230 USD
da Cruz et al., 2014[6]	Romania and Portugal	2010	-	0.186 USD (Romania)<br>0.355 USD (Portugal)
Genc et al., 2019[7]	Turkey	2016	PE, PET, and PP	0.200-0.450 USD

Supplementary Table 3: National plastic recycling rates of research countries and the weighting factors. The import data across four plastic waste types is sourced from the UN Comtrade database from 2013 to 2022. The annual plastic waste is derived by multiplying the annual collected municipal solid waste by the proportion of plastic waste or by direct references. EU country without available plastic waste share is applied with 16%.

Country	Region	Recycling rate	Annual collected municipal solid waste(Mt)	Share of plastic waste	Annual plastic waste (Mt)	Plastic waste imports (PE; 391510; Mt)	Imported plastic waste (PS; 391520; Mt)	Imported plastic waste (PVC; 391530; Mt)	Imported plastic waste (Others; 391590; Mt)
US	North America	5.5% (2021) [8, 9]	292 (2018) [10]	12.2% (2018)	35.6 (2018)	0.61	0.09	0.21	3.29
Canada	North America	9% (2018) [11]	-	-	3 (2020) [12]	0.16	0.05	0.02	0.59
Mexico	North America	32% (2019) [13]	38 (2017) [14]	-	3.8 (2017)	0.13	0.03	0.11	0.43
Netherlands	Western Europe	45.0% (2020) [15]	9 (2021)) [16]	-	1.44 (2021)	3.05	0.08	0.10	3.22
Germany	Western Europe	42.0% (2020) [15]	52 (2021)) [16]	-	8.32 (2021)	2.25	0.27	0.35	2.17
Belgium	Western Europe	39.0% (2020) [15]	8 (2021)) [16]	-	1.28 (2021)	1.31	0.03	0.05	0.76
Spain	Western Europe	43.0% (2020) [15]	22 (2021)) [16]	-	3.52 (2021)	0.41	0.20	0.06	0.61
France	Western Europe	25.0% (2020) [15]	38 (2021)) [16]	-	6.08 (2021)	0.28	0.07	0.14	0.44
Italy	Western Europe	34.0% (2020) [15]	-	-	4.8 (2021) [17]	0.65	0.07	0.02	0.87
United Kingdom	Western Europe	37.0% (2020) [15]	-	-	5 (2018) [18]	0.29	0.01	0.05	0.31

Czech Republic	Eastern Europe	39.0% (2020) [15]	6 (2021)) [16]	16% (2013) [19]	0.96 (2021)	0.04	0.08	0.16	0.72
Slovenia	Eastern Europe	35.0% (2020) [15]	0.85 (2021) [16]	-	0.14 (2021)	0.42	0.00	0.00	0.33
Hong Kong (China)	Rest of Asia	11% (2021) [20]	4 (2021) [21]	21% (2021) [22]	0.08 (2021)	0.75	0.39	0.12	4.48
China	Rest of Asia	30% (2022) [23]	320 (2018) [24]	21% (2020) [25]	67.2 (2008)	16.96	0.70	1.75	18.76
Taiwan (China)	Rest of Asia	26.5% (2021) [26]	11 (2021) [27]	26.28 % (2021) [28]	2.89 (2021)	0.60	0.06	0.27	1.38
India	Rest of Asia	20% (2022) [29]	-	-	3.5 (2021) [30]	0.20	0.00	0.34	0.95
South Korea	Rest of Asia	13.5% (2017) [31]	-	-	12 (2021) [32]	0.09	0.02	0.23	0.51
Turkey	Rest of Asia	13% (2020) [33]	-	-	3.7 (2021) [34]	2.13	0.08	0.02	1.53
Malaysia	Southeast Asia	24% (2019) [35]	-	-	1.07 (2022) [36]	1.97	0.04	0.01	0.62
Indonesia	Southeast Asia	11% (2017) [37]	-	-	6.8 (2021) [38]	0.81	0.00	0.01	0.69
Thailand	Southeast Asia	21% (2018) [39]	-	-	2.8 (2021) [40]	0.21	0.00	0.01	0.75
Vietnam	Southeast Asia	15% (2018) [41]	24 (2019) [42]	7%[43]	1.68 (2019)	0.50	0.01	0.01	1.14

Supplementary Table 4: Countries involved in plastic waste trade from the Global North and the Global South in this work.

Global North	Global South
Andorra. Australia. Austria. Belgium. Bermuda. Canada. Channel Islands. Chile. Croatia. Czechia. Denmark. Estonia. Faeroe Islands. Finland. France. Germany. Greece. Greenland. Hungary. Iceland. Ireland. Isle of Man. Israel. Italy. Japan. Malta. Monaco. Republic of Korea. Latvia. Lithuania. Luxembourg. Mexico. the Netherlands. New Zealand. Norway. Poland. Portugal. San Marino. Slovakia. Slovenia. Spain. Sweden. Switzerland. Turkey. United Kingdom. and the USA	China. China (Taiwan). India. Indonesia. Malaysia. Mexico. Thailand. Turkey. and Vietnam*.

Note: The Global North. as defined by the United Nations Conference on Trade and Development (UNCTAD) [44]. broadly includes Northern America. Europe. Israel. Japan. South Korea. Australia. and New Zealand. The Global South encompasses Africa. Latin America. the Caribbean. and Asia excluding Israel. Japan. South Korea. and Oceania excluding Australia and New Zealand. * Global South countries are not limited to these nine research countries representing leading importers in the plastic waste trade.

Supplementary Table 5: Comparison of the average *RRR* by trade data source and weighting methods. The mass-weighted and value-weighted averages are calculated using the traded plastic waste mass and traded value, respectively. Trade values from 2013 to 2022 have been adjusted to constant 2022 prices using the yearly Consumer Price Index (CPI) of the USA from the World Bank[45].

Trade data source	Weighting method for averaging *RRR*	Average *RRR*	Average *RRR* of plastic PE	Average *RRR* of plastic PS	Average *RRR* of plastic PVC	Average *RRR* of plastic Others
Research countries	Mass-weighted	63%	58%	56%	70%	65%
Research countries	Value-weighted	64%	60%	59%	71%	67%
Trading partners of research countries	Mass-weighted	58%	54%	52%	68%	59%
Trading partners of research countries	Value-weighted	59%	56%	53%	69%	60%

Supplementary Table 6: Estimated share of recycled HDPE and LDPE from recycling PE waste, and PET and PP from recycling plastic waste 'Others'. Regional averages are used when data is unavailable in the research country, with regions defined as in Supplementary Table 3. The recycling share represents the overall plastic waste of a country, including both domestically generated and imported plastic waste. Due to incomplete data availability for each research year, we tested the sensitivity of *RRR* results by assuming recycling PE waste into either HDPE or LDPE exclusively, and recycling plastic waste 'Others' into either PET or PP exclusively. The resulting *RRR* values for PE and 'Others' were subject to average ±5% and ±10% variations, respectively. For detailed results by country and year, please refer to the '*RRR* changes by product range' sheet in the supplementary data.

Country	Share of PET recycling	Share of PP recycling	Share of HDPE recycling	Share of LDPE recycling
Malaysia[46]	29 (2019)	5 (2019)	52 (2019)	8 (2019)
Thailand[47]	15 (2018)	19 (2018)	30 (2018)	26 (2018)
Philippines[48]	14 (2019)	22 (2019)	6 (2019)	11 (2019)
Vietnam (Hanoi) [29]	42 (2022)	-	30 (2022)	1 (2022)
Indonesia (Greater Jakarta) [29]	65 (2020)	11 (2020)	11 (2020)	5 (2020)
Indonesia (Makassar) [29]	70 (2020)	10 (2020)	10 (2020)	5 (2020)
India[29] (Delhi)	14 (2022)	7 (2022)	20 (2022)	25 (2022)
India[29] (Mumbai)	11 (2022)	4 (2022)	22 (2022)	26 (2022)
India[29] (Chennai)	15 (2022)	5 (2022)	20 (2022)	27 (2022)
Hongkong, China[49]	3 (2020)	6 (2020)	10 (2020)	9 (2020)
Taiwan, China[50]	55 (2019)	21 (2019)	-	-
China[23]	33 (2022)	21 (2022)	10 (2022)	12 (2022)
Japan[51]	30 (2021)	23 (2021)	20 (PE in total; 2021)	20 (PE in total; 2021)
United Kingdom[52]	34 (2017)	7 (2017)	26 (2017)	3 (2017)
United States[53]	31 (2017)	2 (2017)	20 (2017)	11 (2017)
Spain[54]	22 (2011)	4 (2011)	24 (2011)	29 (2011)
Germany[55]	13 (2017)	26 (2017)	22 (2017)	20 (2017)
EU27[56]	22 (2019)	16 (2019)	18 (2019)	21 (2019)

Supplementary Table 7: Stepwise changes during data processing in trade entries of plastic waste and primary plastics sourced from the UN Comtrade database.

Data processing	Plastic waste imports (22 research countries)	Plastic waste exports (trading partners of 22 research countries)	Primary plastics exports (22 research countries)	Primary plastics imports (trading partners of 22 research countries)
Trade value originally retrieved from the UN Comtrade database	21940 entries	20802 entries	70311 entries	73808 entries
Dropped Entries with empty net weight or trade value	21884 entries, 56 dropped (0.26% of the original trade)	20721 entries, 81 dropped (0.39% of the original trade)	70135 entries, 176 dropped (0.25% of the original trade)	73378 entries, 430 dropped (0.58% of the original trade)
Drop trade value outliers[1]	21366 entries, 518 dropped (2.36% of the original trade)	20273 entries, 448 dropped (2.15% of the original trade)	68335 entries, 1800 dropped, (2.56% of the original trade)	71427 entries, 1951 dropped, (2.64% of the original trade)
Grouped trade values by research countries, period, and plastic types	880 entries, 24 missing (2.73 % of the grouped entries)	880 entries, 1 missing (0.11% of the grouped entries)	1320 entries, 144 missing (10.91% of the grouped entries)	1320 entries, 33 missing (2.50% of the grouped entries)
Replacing empty trade value (if mirror data exists)[2]	880 entries (23 replaced (2.62%), 1 missing (0.11%))	880 entries (0 replaced (0%), 1 missing (0.11%))	1320 entries (33 replaced (2.50%), 111 missing (8.41%))	1320 entries (0 replaced (0%), 33 missing (2.50%))

Note: * Referring to Chatham House's method in identifying the bilateral trade outliers[57], we assume that the logarithm of the unit price follows a normal distribution. Trade value outliers are identified by their calculated unit price greater than three standard deviations from the mean value. Please refer to the methodology section for further details.

Supplementary Table 8: Annual recycling output and number of employees in plastic recycling companies across countries. The data referring company's annual recycling output and number of employees are voluntarily disclosed on their independent websites, which are publicly accessible (accessed on April 24th, 2024). The person-hours to recycle 1 kg are subsequently averaged at the country level. Data for 'EU 27+3' is applied to nine European research countries plus South Korea and Hong Kong (China). Canada shares the data of the United States.

Coverage	Data sources	References (or links)	Annual recycling output	Number of Employees	Production rate per person-hours (kg/person-hour)	Person-hours to recycle 1 kg
EU27+3	Industry Associations	Plastic Recyclers Europe (2022) [58]	10.2 Mt	30000	116	0.0086
USA	Industry Associations	American Chemistry Council (2019) [59]	6.5 Mt	24000	93	0.0108
USA	Government departments	United States Environmental Protection Agency (2020) [60]	-	-	146	0.0070
USA	Company disclosure	Deltco Plastics (https://www.deltcoplastics.com/)	27.2kt	90	104	0.0096
USA	Company disclosure	Evergreen (https://www.evergreentogether.com/)	67kt	185	124	0.0081
USA	Company disclosure	Peninsula Plastics Recycling (https://peninsularecycling.com/)	27kt	92	101	0.0099
USA	Company disclosure	Atkore Northwest Polymers (https://nwpoly.com/)	18kt	60	103	0.0097
USA	Company disclosure	Resource Plastics Inc. (http://www.resource-plastics.com/)	34kt	100	117	0.0085
Mexico	Company disclosure	Indorama Ventures EcoMex, S de RL de CV (https://www.indoramaventures.com/en/worldwide/816/indorama-ventures-ecomex)	42kt	177	81	0.0120
Mexico	Company disclosure	ALPLA Recycling[61]	15kt	70	73	0.0137

Turkey	Industry Associations	Recycling and Recoverers Association (GEKADER) (https://gekader.org.tr/)	12Mt	70000[a]	59	0.0170
Turkey	Company disclosure	Folyopak Ambalaj San. Ve Tic. Inc. (https://folyopak.com.tr/)	18kt	140	44	0.0230
Turkey	Company disclosure	Hür Plastik Geri Dönüşüm LTİ. STİ. (https://www.hurplastik.com/)	30kt	140	73	0.0140
Turkey	Company disclosure	Plasman Polimer San. Ltd. Şti. (https://www.plasman.com.tr/en/home-2/)	10kt	50	68	0.0150
China	Industry Associations	China Plastic Recycling Association (2022) [62]	16Mt	100000	65	0.0150
China	Company disclosure	Zhejiang Haili Environmental Technology Co., Ltd. (https://www.hailirecycle.com/)	182kt	600	104	0.0096
India	Industry Associations	PlastIndia Foundation (2019) [63]	6Mt	100000[b]	21	0.0476
India	Company disclosure	Ganesha Ecosphere Ltd. (https://ganeshaecosphere.com/)	119kt	1000	41	0.0240
India	Company disclosure	The Shakti Plastic Industries (https://www.shaktiplasticinds.com/)	120kt	500	82	0.0120
Malaysia	Company disclosure	Karich Sdn Bhd (http://www.karich.com.my/plastic-recycling)	15kt	270	19	0.0530
Malaysia	Company disclosure	Dragon Alliance Sdn. Bhd (https://www.daplastics.com/)	36kt	300	41	0.0244
Malaysia	Company disclosure	SG Green Resources Sdn. Bhd (https://sggreen.com.my/index.html)	6kt	70	29	0.0345
Taiwan (China)	Company disclosure	REMONDIS Taiwan (https://www.remondis-taiwan.com.tw/en/about-us/)	40kt	110	125	0.0080
Indonesia	Company disclosure	PT. Pradha Karya Perkasa (https://www.prakarsarecycling.com/)	30kt	320	32	0.0310
Indonesia	Company disclosure	Veolia Indonesia (https://aqua.co.id/en/danone-aqua-and-veolia-	25kt	225	38	0.0260

		indonesia-inaugurate-the-most-modern-and-largest-plastic-recycling-facility-in-indonesia)				
Vietnam	Company disclosure	Công ty TNHH Công Nghiệp và Dịch Vụ Bình Minh (https://abmchemical.com/)	27kt	150	61	0.0160
Vietnam	Company disclosure	Vinatic Hai Phong Company Ltd. (https://www.vinatic.com.vn/pages/abous-us)	60kt	250	82	0.0120
Thailand	Company disclosure	Billion Enterprise. Co., Ltd. (https://www.billionpolymer.com/)	10.8kt	75	49	0.020
Thailand	Company disclosure	Indorama Polyester Industries PCL[64]	57kt	600[c]	33	0.0303

Note: [a] With a total workforce of 350,000, it is assumed that the ratio of direct to indirect employment is 1:4[59]. [b] Together for direct and indirect employment. [c] Workers for polyester plastic production are included.

Supplementary Table 9: Hourly earnings of employees in the manufacturing industry across countries during 2013-2022 (USD$). The data is derived from the dataset of 'Average monthly earnings of employees by sex and economic activity' compiled by the International Labour Organization, given working 8 hours a day and 20 workdays a month.

Country	2013	2014	2015	2016	2017	2018	2019	2020	2021	2022
Belgium	26.94	27.77	23.23	23.27	24.20	25.84	44.49	45.58	47.84	47.84
Canada	29.99	28.85	25.52	24.89	21.58	22.23	22.47	23.21	24.89	25.48
China	2.69	3.02	3.26	3.30	3.47	3.88	3.99	4.37	5.16	5.16
Czech Republic	8.42	8.13	7.15	7.51	8.43	9.77	9.77	10.11	10.11	10.11
France	26.24	24.13	22.65	22.91	24.08	25.66	24.71	16.21	35.21	35.21
Germany	25.72	29.10	24.05	23.53	25.03	27.32	26.59	39.56	38.35	38.35
Hong Kong (China)	10.71	11.53	12.01	12.56	12.91	13.48	14.20	14.10	14.24	14.61
India	1.17	1.17	1.17	1.17	1.17	1.17	1.21	1.23	1.24	1.24
Indonesia	0.87	0.84	0.77	0.98	1.14	1.09	1.17	1.13	1.16	1.18
Italy	26.82	20.97	14.42	14.46	14.87	15.66	14.99	15.41	25.09	25.09
South Korea	18.53	20.86	19.72	19.46	20.22	21.87	21.28	20.71	22.18	21.21
Malaysia	3.84	3.84	3.26	3.54	3.55	3.97	3.95	3.78	3.78	3.78
Mexico	4.55	4.54	3.95	3.53	3.71	3.86	2.24	2.08	2.36	2.59
Netherlands	36.64	27.24	32.10	33.40	35.50	23.33	36.80	38.72	40.55	40.55
Taiwan (China)	9.57	9.70	9.59	9.53	10.42	10.99	10.89	11.48	12.86	12.69
Slovenia	16.13	12.50	10.93	11.10	11.63	12.60	12.35	16.50	17.86	17.86
Spain	18.20	18.14	15.25	14.84	16.01	17.00	19.63	21.32	20.87	20.87
Thailand	2.71	2.60	1.95	2.18	2.18	2.76	2.93	2.90	2.89	2.85
Turkey	3.82	5.62	3.27	3.50	3.18	4.71	4.71	5.03	2.57	2.36
UK	24.79	24.79	24.64	22.62	21.82	23.32	22.73	21.67	25.36	23.65
USA	25.79	25.96	26.70	27.27	28.21	29.04	29.87	31.61	32.26	34.11
Vietnam	1.17	1.28	1.50	1.59	1.51	1.61	1.79	1.92	1.80	2.01

Supplementary Table 10: Electricity consumption per kilogram of plastic waste in plastic mechanical recycling (kWh/kg). The average electricity consumption for each type of plastic waste is applied to the analysis.

References	Coverage	HDPE	LDPE	PS	PVC	PET	PP
Life cycle inventory (Federal LCA Commons) [65]	USA	0.49	-	-	-	0.46	-
Life cycle inventory (Ecoinvent) [66]	Europe	0.49	-	-	-	0.94	-
Life cycle inventory (Ecoinvent) [66]	India	0.21	0.21	-	-	0.21	0.21
Lim, Ahn and Kim [67]	Korea	0.29	0.29	0.29	0.29	0.29	0.29
Shan, Pandyaswargo and Onoda [68]	Japan	0.5	0.5	0.5	0.5	0.5	0.5
Civancik-Uslu, Nhu [69]	Belgium	0.57	0.57	0.50	-	-	0.59
Schwarz, Ligthart [70]	Global	0.47	0.47	0.47	0.47	0.47	0.47
Meys, Frick [71]	Global	0.55	0.76	0.55	-	0.5	0.55
Uekert, Singh [72]	USA	0.43	0.83	-	-	0.43	0.43
Larrain, Van Passel [3]	Belgium	-	0.30	0.25	-	-	0.35
Average	-	0.44	0.49	0.43	0.42	0.48	0.42

Supplementary Table 11: Electricity prices for non-household consumers of European countries during 2013-2022 (USD/kWh). The value is averaged from the original bi-annual data that includes all taxes and levies. The price is adjusted with the yearly average exchange rate of EUR to USD.

Country	2013	2014	2015	2016	2017	2018	2019	2020	2021	2022
Slovenia	0.154	0.139	0.115	0.113	0.108	0.124	0.131	0.137	0.138	0.220
Czech Republic	0.162	0.132	0.104	0.098	0.096	0.104	0.105	0.117	0.124	0.213
Germany	0.251	0.270	0.219	0.218	0.225	0.233	0.225	0.249	0.268	0.271
Netherlands	0.152	0.147	0.117	0.111	0.108	0.119	0.125	0.145	0.169	0.213
Spain	0.195	0.194	0.155	0.143	0.143	0.154	0.153	0.156	0.178	0.256
Belgium	0.175	0.172	0.144	0.152	0.150	0.159	0.155	0.162	0.189	0.274
Italy	0.262	0.268	0.207	0.198	0.191	0.195	0.211	0.199	0.235	0.362
France	0.145	0.153	0.132	0.123	0.129	0.132	0.132	0.137	0.146	0.158
United Kingdom	0.187	0.210	0.200	0.176	0.169	0.195	0.206	0.225	-	-

Supplementary Table 12: Electricity prices for non-household consumers of other research countries during 2013-2022 (USD/kWh). The value is averaged from the country's industrial electricity tariff. The missing value is filled by the nearest valid observations in the same country. The USD price is adjusted with the yearly average exchange rate of local currency to USD.

Country	Year	Local price	Currency	USD/kWh	Issuing authorities, policies, and effective time
Malaysia	2013	0.312	MYR	0.099	Tenaga Nasional Berhad (2011) [73]
Malaysia	2014	0.365	MYR	0.112	Tenaga Nasional Berhad (2014) [54]
Malaysia	2015	0.365	MYR	0.089	
Malaysia	2016	0.365	MYR	0.089	
Malaysia	2017	0.365	MYR	0.087	
Malaysia	2018	0.365	MYR	0.091	
Malaysia	2019	0.365	MYR	0.090	
Malaysia	2020	0.365	MYR	0.088	
Malaysia	2021	0.365	MYR	0.095	
Malaysia	2022	0.365	MYR	0.081	
Indonesia	2013	796.35	IDR	0.076	Decree No. 1404 K/20/MEM/2017[74]
Indonesia	2014	977.77	IDR	0.082	Decree No. 1772 K/20/MEM/2018[74]
Indonesia	2015	1142.72	IDR	0.085	Decree No. 55 K/20/MEM/2019[75]
Indonesia	2016	1051.82	IDR	0.079	Decree No. 169.K/HK.02/MEM.M/2021[75]
Indonesia	2017	1088.8	IDR	0.081	
Indonesia	2018	1085.3	IDR	0.076	
Indonesia	2019	1100.69	IDR	0.078	
Indonesia	2020	1090.89	IDR	0.075	
Indonesia	2021	1086.22	IDR	0.076	
Indonesia	2022	1080.66	IDR	0.073	
Vietnam	2013	1378	VND	0.066	Decision No. 268/QD-TTg (2011) [76]
Vietnam	2014	1514	VND	0.072	Decision No. 28/2014/QD-TTg (2014) [77]
Vietnam	2015	1514	VND	0.069	Ref. 648/QD-BCT (2019) [78]
Vietnam	2016	1514	VND	0.068	
Vietnam	2017	1514	VND	0.067	
Vietnam	2018	1514	VND	0.066	
Vietnam	2019	1611	VND	0.069	
Vietnam	2020	1611	VND	0.070	
Vietnam	2021	1611	VND	0.070	
Vietnam	2022	1611	VND	0.069	
Thailand	2013	3.18	THB	0.103	Provincial electricity authority (2015) [79]
Thailand	2014	3.18	THB	0.098	Provincial electricity authority (2018) [80]
Thailand	2015	3.18	THB	0.093	
Thailand	2016	3.18	THB	0.090	
Thailand	2017	3.18	THB	0.094	
Thailand	2018	3.15	THB	0.097	

Thailand	2019	3.15	THB	0.102	
Thailand	2020	3.15	THB	0.101	
Thailand	2021	3.15	THB	0.099	
Thailand	2022	3.15	THB	0.090	
India	2013	-	INR	-	Tariff Schedule for FY 2014-15[81]<br>Tariff Schedule for FY 2015-16[82]<br>Tariff Schedule for FY 2017-18[83]<br>Tariff Schedule for FY 2018-19[84]<br>Tariff Schedule for FY 2019-20[85]<br>Tariff Schedule for FY 2020-21[86]<br>Tariff Schedule for FY 2021-22[87]
India	2014	7.6	INR	0.125	
India	2015	7.9	INR	0.123	
India	2016	-	INR	-	
India	2017	7.1	INR	0.109	
India	2018	7.25	INR	0.106	
India	2019	7.75	INR	0.110	
India	2020	7.75	INR	0.105	
India	2021	7.75	INR	0.105	
India	2022	-	INR	-	
China	2013	0.65	CNY	0.106	National Electricity Price Regulatory Notice 2013-2014[88]<br>National Electricity Price Regulatory Notice 2015[89]<br>National Electricity Price Regulatory Notice 2016[90]<br>National Electricity Price Regulatory Notice 2017[91]<br>National Electricity Price Regulatory Notice 2018[92]
China	2014	0.66	CNY	0.107	
China	2015	0.64	CNY	0.102	
China	2016	0.65	CNY	0.098	
China	2017	0.6	CNY	0.089	
China	2018	0.59	CNY	0.089	
China	2019	-	CNY	-	
China	2020	-	CNY	-	
China	2021	-	CNY	-	
China	2022	0.449	CNY	0.067	National Energy Administration (2022) [93]
Hong Kong (China)	2013	1.070	HKD	0.138	CLP Power Hong Kong Limited (2013) [94]<br>CLP Power Hong Kong Limited (2018) [95]<br>CLP Power Hong Kong Limited (2021) [96]
Hong Kong (China)	2014	1.108	HKD	0.143	
Hong Kong (China)	2015	1.142	HKD	0.147	
Hong Kong (China)	2016	1.132	HKD	0.146	
Hong Kong (China)	2017	1.132	HKD	0.145	
Hong Kong (China)	2018	1.177	HKD	0.150	
Hong Kong (China)	2019	1.188	HKD	0.152	
Hong Kong (China)	2020	1.218	HKD	0.157	
Hong Kong (China)	2021	1.218	HKD	0.157	
Hong Kong (China)	2022	1.289	HKD	0.165	
Taiwan (China)	2013	2.875	TWD	0.097	Taiwan Power Company (2013-2020) [97]
Taiwan (China)	2014	3.12	TWD	0.103	
Taiwan (China)	2015	2.9	TWD	0.091	

Taiwan (China)	2016	2.625	TWD	0.081	
Taiwan (China)	2017	2.55	TWD	0.084	
Taiwan (China)	2018	2.625	TWD	0.087	
Taiwan (China)	2019	2.63	TWD	0.085	
Taiwan (China)	2020	2.625	TWD	0.089	
Taiwan (China)	2021	-	TWD	-	
Taiwan (China)	2022	-	TWD	-	
South Korea	2013	94	KRW	0.086	S&P Global (2021) [98]<br>Korea Electric Power Corporation (2021) [99]
South Korea	2014	94	KRW	0.089	
South Korea	2015	94	KRW	0.083	
South Korea	2016	94	KRW	0.081	
South Korea	2017	94	KRW	0.083	
South Korea	2018	94	KRW	0.085	
South Korea	2019	94	KRW	0.081	
South Korea	2020	94	KRW	0.080	
South Korea	2021	97	KRW	0.085	
South Korea	2022	97	KRW	0.075	
Turkey	2013	0.238	TRY	0.125	Turkish Statistical Institute (1st half 2013) [100]<br>Turkish Statistical Institute (2nd half 2013) [101]<br>Turkish Statistical Institute (1st half 2014) [102]<br>Turkish Statistical Institute (2nd half 2014) [103]<br>Turkish Statistical Institute (1st half 2015) [104]<br>Turkish Statistical Institute (2nd half 2015) [105]<br>Turkish Statistical Institute (1st half 2016) [106]<br>Turkish Statistical Institute (2nd half 2016) [107]<br>Turkish Statistical Institute (1st half 2017) [108]<br>Turkish Statistical Institute (2nd half 2017) [109]<br>Turkish Statistical Institute (1st half 2018) [110]<br>Turkish Statistical Institute (2nd half 2018) [111]<br>Turkish Statistical Institute (1st half 2019) [112]<br>Turkish Statistical Institute (2nd half 2019) [113]<br>Turkish Statistical Institute (1st half 2020) [114]<br>Turkish Statistical Institute (2nd half 2020) [115]<br>Turkish Statistical Institute (1st half 2021) [116]<br>Turkish Statistical Institute (2nd half 2021) [117]<br>Turkish Statistical Institute (1st half 2022) [118]
Turkey	2014	0.235	TRY	0.107	
Turkey	2015	0.244	TRY	0.089	
Turkey	2016	0.252	TRY	0.083	
Turkey	2017	0.258	TRY	0.071	
Turkey	2018	0.372	TRY	0.077	
Turkey	2019	0.514	TRY	0.091	
Turkey	2020	0.58	TRY	0.082	
Turkey	2021	0.85	TRY	0.096	
Turkey	2022	0.809	TRY	0.049	
USA	2013	0.068	USD	0.068	IEA (2013) [119]
USA	2014	0.071	USD	0.071	
USA	2015	0.069	USD	0.069	

USA	2016	0.068	USD	0.068	
USA	2017	0.069	USD	0.069	
USA	2018	0.068	USD	0.068	
USA	2019	0.067	USD	0.067	
USA	2020	0.066	USD	0.066	
USA	2021	0.069	USD	0.069	
USA	2022	0.084	USD	0.084	
Canada	2013	0.081	USD	0.081	IEA (2013) [119]
Canada	2014	0.072	USD	0.072	
Canada	2015	0.066	USD	0.066	
Canada	2016	0.087	USD	0.087	
Canada	2017	0.09	USD	0.090	
Canada	2018	0.091	USD	0.091	
Canada	2019	0.1	USD	0.100	
Canada	2020	-	USD	-	
Canada	2021	-	USD	-	
Canada	2022	-	USD	-	
Mexico	2013	-	USD	-	NERA Economic Consulting (2016-2019) [120] National Centre for Energy Control (2020-2021) [121]
Mexico	2014	-	USD	-	
Mexico	2015	-	USD	-	
Mexico	2016	0.047	USD	0.047	
Mexico	2017	0.066	USD	0.066	
Mexico	2018	0.083	USD	0.083	
Mexico	2019	0.073	USD	0.073	
Mexico	2020	0.025	USD	0.025	
Mexico	2021	0.028	USD	0.028	
Mexico	2022	-	USD	-	

Supplementary Table 13: Annual recycling output and land occupation in plastic recycling companies across countries. Except for land information voluntarily disclosed by companies, other data on land occupation is roughly measured by Google Earth[122]. The recycling output data is voluntarily disclosed by companies on their websites without third-party inspection. The data is subsequently averaged by countries. Data from Germany is also applied to the Netherlands and South Korea where data is missing. Slovenia and Canada share the data of the Czech Republic and the USA, respectively.

Region	Plastic mechanical recycling company (link to company website)	Recycling output (tonne/yr)	Land occupation (m^2)	m^2/tonne*yr
UK	Roydon Bottle Recycling Ltd	17500	12000	0.686
UK	PlasRecycle Ltd	20000	9290	0.465
UK	MBA Polymers UK Ltd	60000	52650	0.878
Belgium	Tivaco NV/SA	22000	10000	0.455
Czech Republic	Remaq. s.r.o.	28800	16500	0.573
Spain	Plásticos Güell. SL	14500	7300	0.503
Spain	Loginplast. S.L.U.	50000	28000	0.560
Spain	Iber Resinas SL	48000	50000	1.042
Spain	Green World Compounding SL	100000	100000	1.000
Italy	Braghieri Plastic S.r.l.	4250	2800	0.659
Italy	A.M.P. Recycling S.r.l.	60000	52000	0.867
France	Sarl Sedem	12000	7280	0.607
France	Nord Pal Plast	11000	14000	1.273
France	Galloo Plastics SA	90000	31500	0.350
Germany	Kunststoff Recycling Grünstadt GmbH	50000	32000	0.640
Germany	Best Plastic Management GmbH	20000	17000	0.850
Germany	Copo Plast Kunststoffrecycling Heinrich Winkler e.K.	20000	15000	0.750
USA	Evergreen	98600	23225	0.236
USA	Peninsula Plastics Recycling	27000	16722	0.619
USA	Atkore Northwest Polymers	20000	15300	0.765
USA	Resource Plastics Inc.	34000	16300	0.479
Mexico	Omnigreen SAPI de CV	28800	20500	0.712
Mexico	Alcamare. S. de R.L. de C.V.	75000	46000	0.613
Mexico	Indorama Ventures EcoMex. S de RL de CV	42000	21200	0.505
Turkey	Ard Geri Dönüşüm Bilişim Plastik İnşaat Nakliye San. Ve. Ltd. Şti.	3600	2200	0.611
Turkey	Boliş Plastik ve Kimya San. Tic. Ltd. Şti.	9600	3500	0.365
Turkey	Garanti Geri Dönüşüm	24000	15000	0.625
Turkey	Hür Plastik Geri Dönüşüm ve Granül imalatı San. Tic. Ltd. Şti	30000	28000	0.933
Hong Kong (China)	New Life Plastics Ltd.	35000	6500	0.186

China	Shanghai Tianqiang Environmental Protection Technology Co.. Ltd.	50000	15000	0.300
China	Jiangxi Royal One Renewable Resources Co.. Ltd.	80000	42000	0.525
India	Al Mehtab Industries Pvt. Ltd	26000	22296	0.858
India	The Shakti Plastic Industries	120000	80940	0.675
Malaysia	Plasticycle Industries Sdn Bhd	8000	9500	1.188
Malaysia	Fizlestari Plastic Sdn Bhd	10000	15000	1.500
Malaysia	Karich Sdn Bhd	15000	17440	1.163
Taiwan (China)	Ming Chuan Plastic Co. Ltd.	15000	5000	0.333
Taiwan (China)	REMONDIS Taiwan	40000	15500	0.388
Indonesia	Langgeng Jaya Group	72000	32000	0.444
Indonesia	PT. Pradha Karya Perkasa	36000	42000	1.167
Indonesia	Veolia Indonesia	25000	22000	0.880
Vietnam	Công ty TNHH Công Nghiệp và Dịch Vụ Bình Minh	27000	20000	0.741
Vietnam	Vinatic Hai Phong Company Ltd.	60000	60000	1.000
Thailand	Billion Enterprise. Co.. Ltd.	10800	10800	1.000
Thailand	Thai Plastic Recycle Group Co.. Ltd.	25000	25000	1.000

Supplementary Table 14: Industrial rents across countries during 2013–2022 (USD/m^2). In some instances, the data represents rents in key industrial areas rather than national averages. For example, China's data is based on rents in industrial areas of Shanghai. Additionally, 'py' (Pyeong) is a Korean unit of area equivalent to 3.3 square meters (35.6 square feet). Data from Germany has been used to represent France, Spain, Italy, the Czech Republic, and Belgium where specific data was unavailable.

Country	Year	Average local price	Unit	Annual rent (USD)/m^2	Publisher and effective time
Thailand	2013	-	-	-	Thailand board of investment (2014) [123]
Thailand	2014	140	THB/m^2/month	51.780	Thailand board of investment (2015) [124]
Thailand	2015	140	THB/m^2/month	49.209	Thailand board of investment (2016) [125]
Thailand	2016	140	THB/m^2/month	47.606	Thailand board of investment (2017) [126]
Thailand	2017	140	THB/m^2/month	49.535	Thailand board of investment (2018) [127]
Thailand	2018	140	THB/m^2/month	51.983	Thailand board of investment (2019) [128]
Thailand	2019	140	THB/m^2/month	54.166	Thailand board of investment (2021) [129]
Thailand	-	-	-	-	Thailand board of investment (2022) [130]
Thailand	2021	150	THB/m^2/month	56.415	
Thailand	2022	150	THB/m^2/month	51.365	
Indonesia	2013	55000	IDR/m^2/month	63.252	Cushman & Wakefield (2013-2020) [131]
Indonesia	2014	60000	IDR/m^2/month	60.739	
Indonesia	2015	70000	IDR/m^2/month	62.676	
Indonesia	2016	70000	IDR/m^2/month	63.161	
Indonesia	2017	70000	IDR/m^2/month	62.773	
Indonesia	2018	70000	IDR/m^2/month	59.036	
Indonesia	2019	70000	IDR/m^2/month	59.385	
Indonesia	2020	70000	IDR/m^2/month	57.701	
Indonesia	2021	-	-	-	
Indonesia	2022	-	-	-	
Malaysia	2013	-	-	-	Cushman & Wakefield (2016-2022) [132]
Malaysia	2014	-	-	-	
Malaysia	2015	-	-	-	
Malaysia	2016	1.5	MYR/ft^2/month	47.183	
Malaysia	2017	1.5	MYR/ft^2/month	45.966	

Malaysia	2018	1.5	MYR/ft^2/month	48.054	
Malaysia	2019	1.5	MYR/ft^2/month	47.720	
Malaysia	2020	1.5	MYR/ft^2/month	46.475	
Malaysia	2021	1.8	MYR/ft^2/month	60.497	
Malaysia	2022	2	MYR/ft^2/month	57.127	
Vietnam	2013	70000	VND/m^2/month	40.162	Kizuna (2013) [133]
Vietnam	2014	70000	-	39.716	
Vietnam	2015	70000	-	38.360	
Vietnam	2016	70000	-	37.582	
Vietnam	2017	70000	-	37.056	
Vietnam	2018	70000	-	36.636	
Vietnam	2019	70000	-	36.158	
Vietnam	2020	70000	-	36.344	
Vietnam	2021	70000	-	36.639	
Vietnam	2022	70000	-	35.938	
India	2013	-	-	-	Cushman & Wakefield (1st half of 2018) [134]
India	2014	-	-	-	
India	2015	-	-	-	Cushman & Wakefield (2nd half of 2019) [135]
India	2016	-	-	-	
India	2017	-	-	-	Cushman & Wakefield (2nd half of 2021) [136]
India	2018	19	INR/ft^2/month	35.912	Cushman & Wakefield (2nd half of 2022) [137]
India	2019	22	INR/ft^2/month	40.340	
India	2020	-	-	-	
India	2021	19	INR/ft^2/month	33.206	
India	2022	22	INR/ft^2/month	36.178	
China	2013	329	CNY/m^2/year	53.526	Knight Frank (2013-2020) [138]
China	2014	347	CNY/m^2/year	56.333	Savills (2022) [139]
China	2015	365	CNY/m^2/year	58.072	
China	2016	372	CNY/m^2/year	56.000	
China	2017	402	CNY/m^2/year	59.538	
China	2018	438	CNY/m^2/year	66.263	
China	2019	456	CNY/m^2/year	66.007	
China	2020	475	CNY/m^2/year	68.903	
China	2021	528	CNY/m^2/year	81.867	

China	2022	528	CNY/m^2/year	78.561	
Hong Kong (China)	2013	9.5	HKD/m^2/month	158.248	CBRE (2013) [140] Knight Frank (2021) [141]
Hong Kong (China)	2014	9.74	HKD/m^2/month	162.295	
Hong Kong (China)	2015	9.98	HKD/m^2/month	166.342	
Hong Kong (China)	2016	10.21	HKD/m^2/month	169.923	
Hong Kong (China)	2017	10.45	HKD/m^2/month	173.230	
Hong Kong (China)	2018	10.69	HKD/m^2/month	176.123	
Hong Kong (China)	2019	10.93	HKD/m^2/month	180.183	
Hong Kong (China)	2020	11.16	HKD/m^2/month	185.894	
Hong Kong (China)	2021	11.4	HKD/m^2/month	189.485	
Hong Kong (China)	2022	11.4	HKD/m^2/month	188.087	
Taiwan (China)	2013	-	-	-	Taiwan Industrial Development Bureau[142]
Taiwan (China)	2014	-	-	-	
Taiwan (China)	2015	-	-	-	
Taiwan (China)	2016	-	-	-	
Taiwan (China)	2017	-	-	-	
Taiwan (China)	2018	-	-	-	
Taiwan (China)	2019	-	-	-	
Taiwan (China)	2020	-	-	-	
Taiwan (China)	2021	-	-	-	
Taiwan (China)	2022	450	TWD/m^2/month	181.390	

South Korea	2013	25550	KRW/py/month	84.613	DWS Group (2013-2019) [143]
South Korea	2014	25550	KRW/py/month	88.010	Real Estate Asia (2021) [144]
South Korea	2015	27000	KRW/py/month	86.427	
South Korea	2016	26700	KRW/py/month	83.434	
South Korea	2017	27600	KRW/py/month	88.532	
South Korea	2018	29700	KRW/py/month	97.890	
South Korea	2019	29600	KRW/py/month	92.035	
South Korea	2020	29500	KRW/py/month	90.787	
South Korea	2021	29487	KRW/py/month	93.370	
South Korea	2022	29487	KRW/py/month	82.883	
Turkey	2013	4.29	USD/m^2/month	51.483	Cushman & Wakefield (2016. 2020. and 2021) [145]
Turkey	2014	4.29	USD/m^2/month	51.478	Cushman & Wakefield (2022) [146]
Turkey	2015	4.29	USD/m^2/month	51.480	
Turkey	2016	4.29	USD/m^2/month	51.479	
Turkey	2017	3.7	USD/m^2/month	44.400	
Turkey	2018	3.7	USD/m^2/month	44.398	
Turkey	2019	3.7	USD/m^2/month	44.402	
Turkey	2020	3.7	USD/m^2/month	44.400	
Turkey	2021	4.1	USD/m^2/month	49.199	
Turkey	2022	5	USD/m^2/month	60.002	
USA	2013	5	USD/m^2/year	40.527	Cushman & Wakefield (2014-2018) [147]
USA	2014	5	USD/ ft^2/year	40.511	Cushman & Wakefield (2019-2022) [148]
USA	2015	5.2	USD/ft^2/year	50.450	
USA	2016	5.4	USD/ft^2/year	52.512	
USA	2017	5.6	USD/ft^2/year	53.360	
USA	2018	5.8	USD/ft^2/year	52.862	
USA	2019	6	USD/ft^2/year	57.694	
USA	2020	6.2	USD/ft^2/year	58.429	
USA	2021	6.4	USD/ft^2/year	58.248	
USA	2022	7.4	USD/ft^2/year	75.649	
Canada	2013	5.5	CAN/ft^2/year	53.396	CBRE (2013-2022) [149]
Canada	2014	6	CAN/ft^2/year	55.214	
Canada	2015	6.2	CAN/ft^2/year	48.049	
Canada	2016	6.5	CAN/ft^2/year	48.652	

Canada	2017	6.5	CAN/ft^2/year	51.528	
Canada	2018	7.26	CAN/ft^2/year	57.167	
Canada	2019	8.69	CAN/ft^2/year	67.042	
Canada	2020	9.58	CAN/ft^2/year	74.087	
Canada	2021	10.47	CAN/ft^2/year	85.107	
Canada	2022	13.71	CAN/ft^2/year	106.402	
Mexico	2013	-	-	-	Cushman & Wakefield (2016-2019) [150]
Mexico	2014	-	-	-	Cushman & Wakefield (2020-2022) [151]
Mexico	2015	-	-	-	
Mexico	2016	0.35	USD/ ft^2/month	45.210	
Mexico	2017	0.35	USD/ ft^2/month	45.210	
Mexico	2018	0.36	USD/ ft^2/month	46.502	
Mexico	2019	0.35	USD/ ft^2/month	45.210	
Mexico	2020	0.37	USD/ ft^2/month	47.793	
Mexico	2021	0.39	USD/ ft^2/month	50.377	
Mexico	2022	0.37	USD/ ft^2/month	47.793	
Germany	2013	-	-	-	Colliers International (2015-2019) [152]
Germany	2014	-	-	-	Jones Lang LaSalle (2018-2022) [153]
Germany	2015	5.6	EUR/m^2/month	74.558	
Germany	2016	5.7	EUR/m^2/month	75.712	
Germany	2017	5.7	EUR/m^2/month	77.271	
Germany	2018	6	EUR/m^2/month	85.032	
Germany	2019	6.3	EUR/m^2/month	84.634	
Germany	2020	6.3	EUR/m^2/month	86.350	
Germany	2021	6.5	EUR/m^2/month	92.251	
Germany	2022	7.5	EUR/m^2/month	94.770	
Netherlands	2013	5.2	EUR/m^2/month	82.873	NAI Netherlands (2013-2019) [154]
Netherlands	2014	5.1	EUR/m^2/month	81.304	
Netherlands	2015	5.3	EUR/m^2/month	70.564	
Netherlands	2016	5.5	EUR/m^2/month	73.055	
Netherlands	2017	5.8	EUR/m^2/month	78.627	
Netherlands	2018	5.9	EUR/m^2/month	83.615	
Netherlands	2019	6	EUR/m^2/month	80.604	
Netherlands	2020	6.1	EUR/m^2/month	83.609	

Netherlands	2021	6.2	EUR/m^2/month	87.993	
Netherlands	2022	6.3	EUR/m^2/month	79.607	
Slovenia	2013	-	-	-	Europe Real Estate (2015) [155]
Slovenia	2014	-	-	-	RE/MAX Commercial (2018-2020) [156]
Slovenia	2015	4.5	EUR/m^2/month	59.913	Colliers International (2021) [157]
Slovenia	2016	-	-	-	Colliers International (2022) [158]
Slovenia	2017	-	-	-	
Slovenia	2018	5.2	EUR/m^2/month	73.694	
Slovenia	2019	4.4	EUR/m^2/month	59.110	
Slovenia	2020	4.9	EUR/m^2/month	67.161	
Slovenia	2021	5.5	-	78.058	
Slovenia	2022	6	-	75.816	
UK	2013	9.071	GBP/ft^2/year	152.734	Knight Frank (2013-2022) [159]
UK	2014	9.165	GBP/ft^2/year	162.668	
UK	2015	9.638	GBP/ft^2/year	158.578	
UK	2016	10.016	GBP/ft^2/year	146.093	
UK	2017	10.394	GBP/ft^2/year	144.263	
UK	2018	10.866	GBP/ft^2/year	156.108	
UK	2019	11.433	GBP/ft^2/year	157.133	
UK	2020	11.622	GBP/ft^2/year	160.725	
UK	2021	11.811	GBP/ft^2/year	174.904	
UK	2022	12.000	GBP/ft^2/year	159.548	

Supplementary Table 15: Physical loss by type during plastic waste mechanical recycling (%). We assume the physical loss for each plastic type remained constant during the research period due to the stable physical properties observed in mechanical recycling processes[160, 161]. Furthermore, the sensitivity analysis considering the impact of physical loss uncertainty on *RRR* values is provided in Fig. 4 and Supplementary Fig. 7–13.

References	Coverage	HDPE	LDPE	PS	PVC	PET	PP
Arena, Mastellone and Perugini [162]	Global	0.24	0.24	0.24	0.24	0.24	0.24
Civancik-Uslu, Nhu [69]	Belgium	0.19	0.19	0.11	-	-	0.15
Brouwer, Picuno [163]	Netherlands (separate collection)	0.08	0.08	-	-	0.12	0.24
Brouwer, Picuno [163]	Netherlands (MSW)	0.20	0.20	-	-	0.15	0.27
Faraca and Astrup [164]	Denmark	-	-	-	0.12	-	-
Plinke, Wenk [165]	Europe	-	-	-	0.10	-	-
Larrain, Van Passel [3]	Belgium	-	0.2	0.13	-	-	0.13
Uekert, Singh [72]	USA	0.17	0.28	-	-	0.26	0.17

2. Supplementary Figures

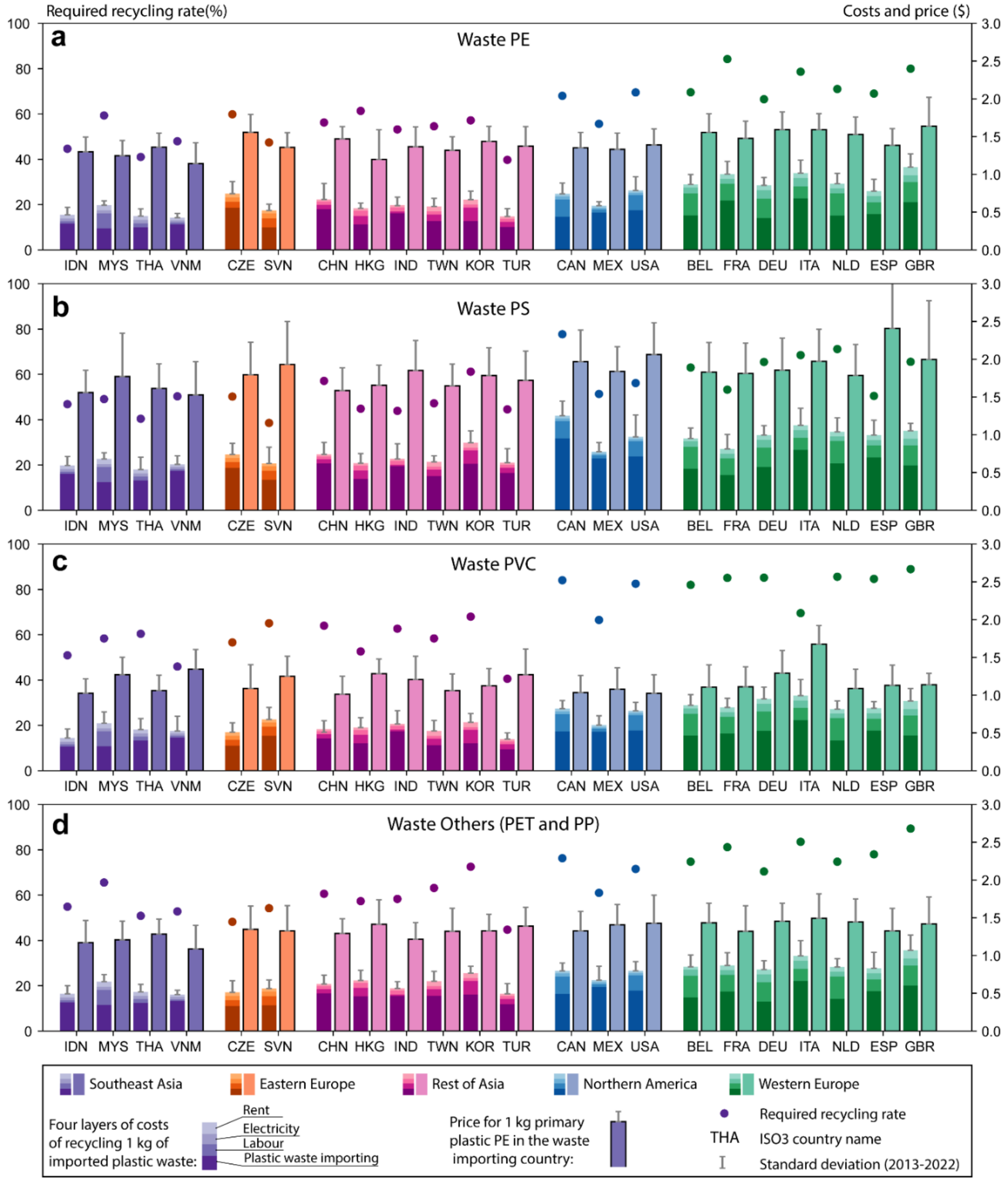

Supplementary Figure 1: Required recycling rate of imported plastic waste by country and plastic waste type (mirror trade data). The *RRR* is displayed for waste PE (a), waste PS (b), waste PVC (c), and waste 'Others' (d). Left bar: Costs related to 1 kg recycled plastic output. Right bar: the value of 1 kg primary plastic. Dot with %: required recycling rate to break even. All the costs and product prices are represented by recycling 1 kg of imported plastic waste.

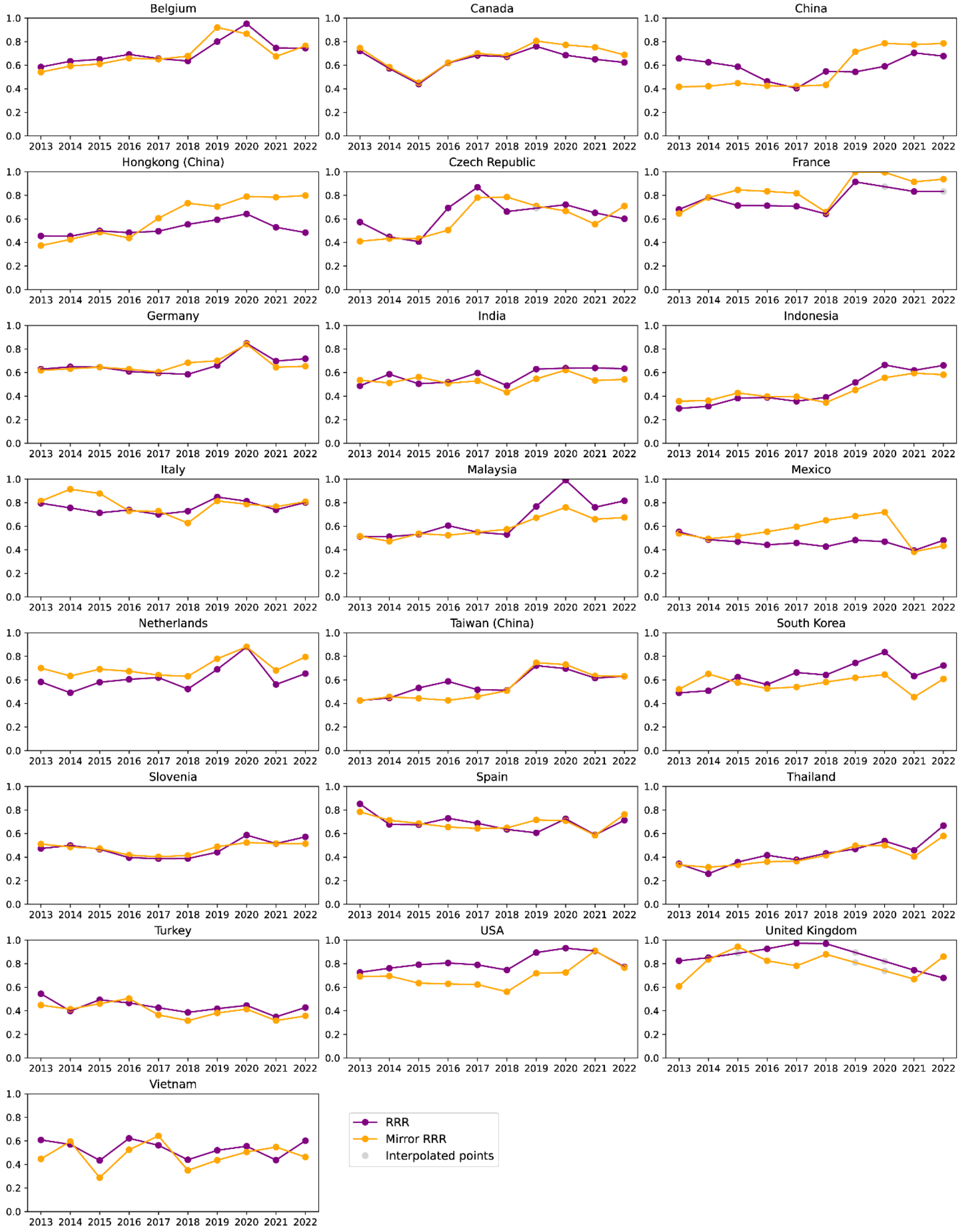

Supplementary Figure 2: Annual *RRR* of plastic waste PE from 2013 to 2022 in 22 research countries. The *RRR* (purple line) and mirror *RRR* (orange line) were calculated using trade data (plastic waste imports and primary plastics exports reported by the 22 research countries) and mirror trade data (plastic waste exports and primary plastics imports reported by the trading partners of the 22 research countries). Missing annual *RRR* values were linearly interpolated and are shown in grey.

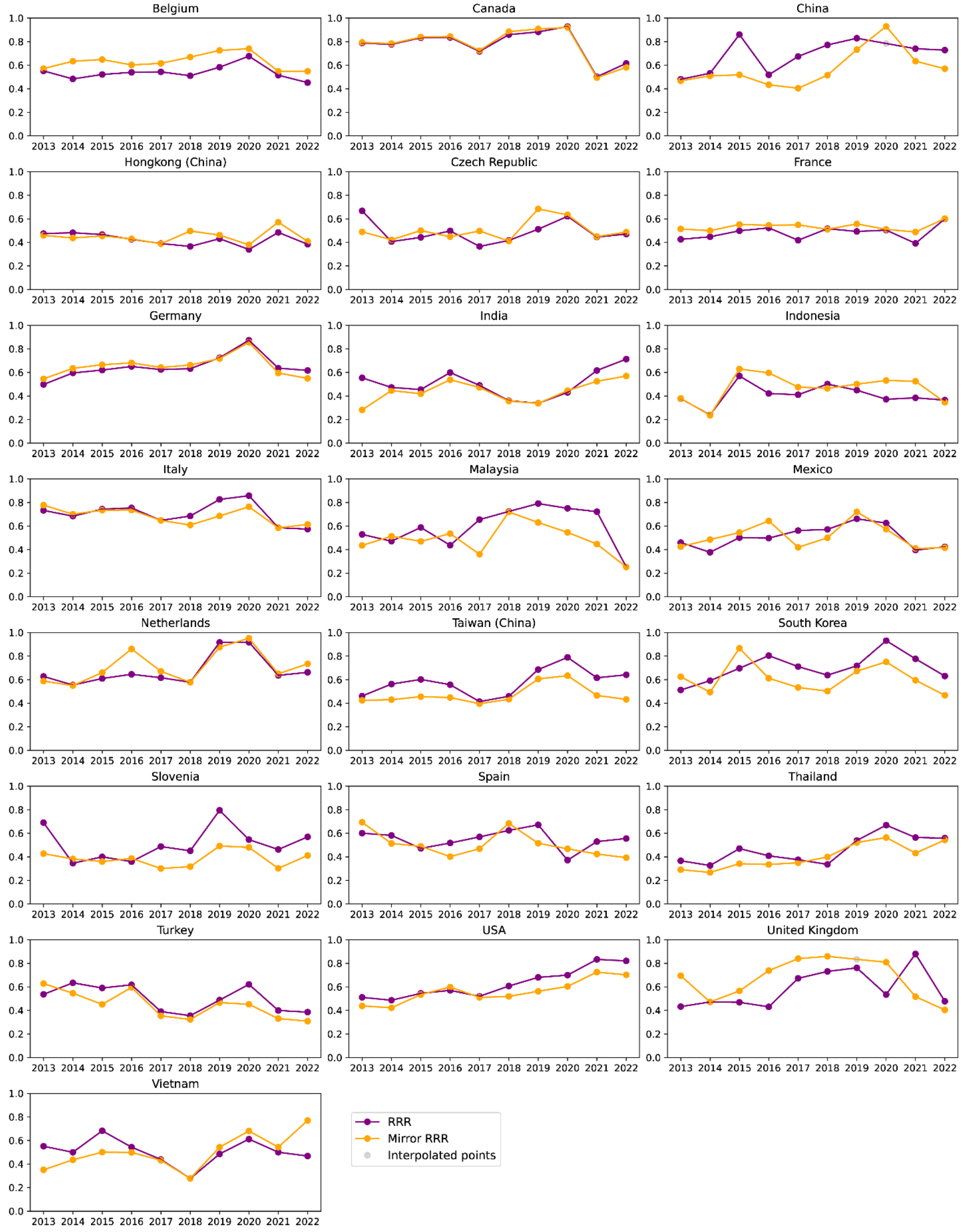

Supplementary Figure 3: Annual *RRR* of plastic waste PS from 2013 to 2022 in 22 research countries. The *RRR* (purple line) and mirror *RRR* (orange line) were calculated using trade data (plastic waste imports and primary plastics exports reported by the 22 research countries) and mirror trade data (plastic waste exports and primary plastics imports reported by the trading partners of the 22 research countries). Missing annual *RRR* values were linearly interpolated and are shown in grey.

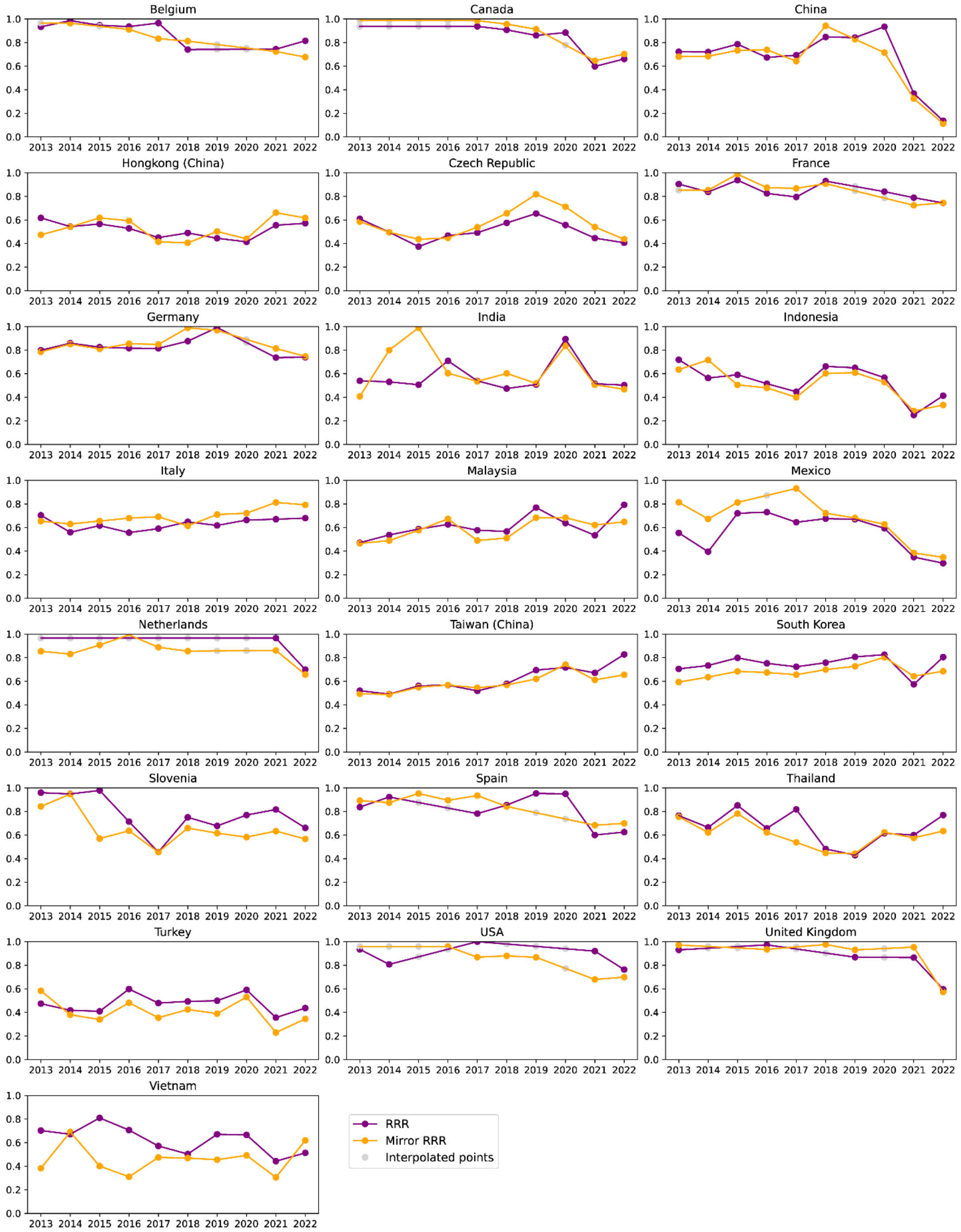

Supplementary Figure 4: Annual *RRR* of plastic waste PVC from 2013 to 2022 in 22 research countries. The *RRR* (purple line) and mirror *RRR* (orange line) were calculated using trade data (plastic waste imports and primary plastics exports reported by the 22 research countries) and mirror trade data (plastic waste exports and primary plastics imports reported by the trading partners of the 22 research countries). Missing annual *RRR* values were linearly interpolated and are shown in grey.

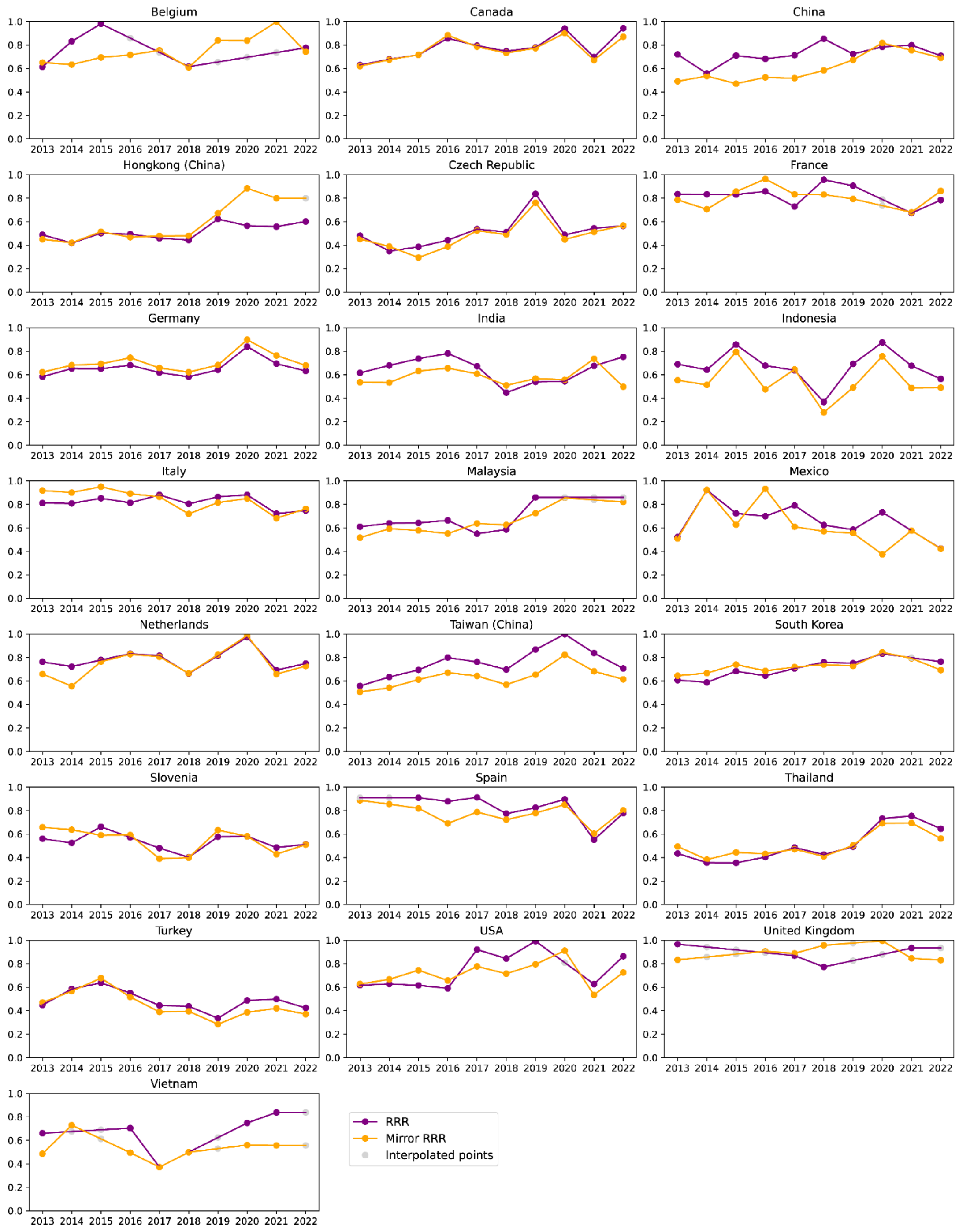

Supplementary Figure 5: Annual *RRR* of plastic waste 'Others' from 2013 to 2022 in 22 research countries. The *RRR* (purple line) and mirror *RRR* (orange line) were calculated using trade data (plastic waste imports and primary plastics exports reported by the 22 research countries) and mirror trade data (plastic waste exports and primary plastics imports reported by the trading partners of the 22 research countries). Missing annual *RRR* values were linearly interpolated and are shown in grey.

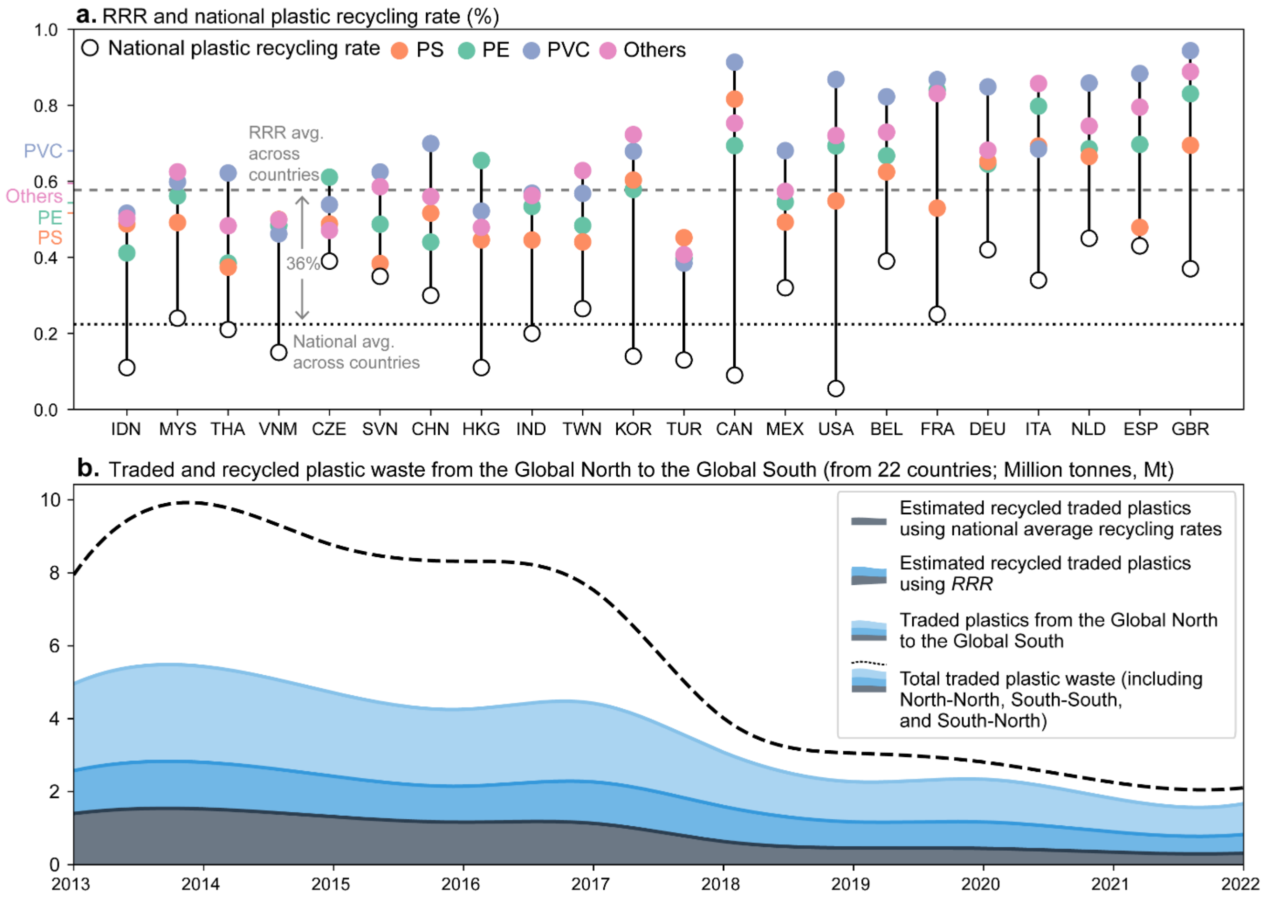

Supplementary Figure 6: The difference between required recycling rates and national plastic recycling rates across 22 countries (mirror trade data). (a) illustrates the variations in average *RRR* across countries and plastic types. The *RRR* was calculated with the mirror trade data including plastic waste exports and primary plastics imports reported by the trading partners of the 22 research countries. (b) shows how these differences influence the estimates of recycling for plastic waste traded from the Global North to the Global South between 2013-2022 (full country names shown in Supplementary Table 4). The trade data used in (b) consists of the mirror trade data including plastic waste exports reported by waste-exporting countries, which originate from the UN Comtrade database.

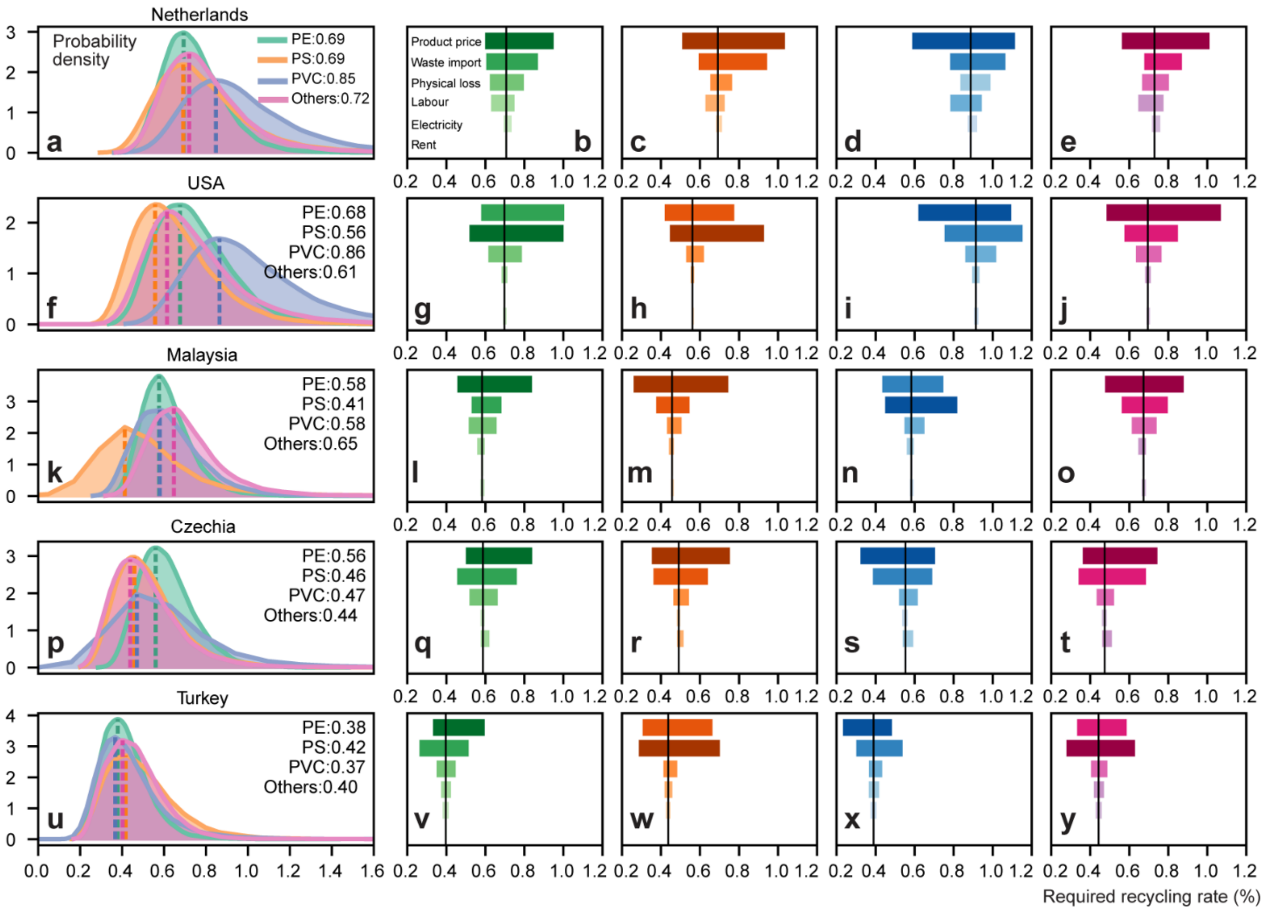

Supplementary Figure 7: Sensitivity analysis and Monte Carlo simulation for the calculated *RRR* (mirror trade data) of six selected countries. The selected countries include the Netherlands (a–e), the USA (f–j), Malaysia (k–o), the Czech Republic (p–t), and Turkey (u–y). The length and colour depth of the horizontal bars are proportional to the range of sensitivity results derived from pessimistic and optimistic cases.

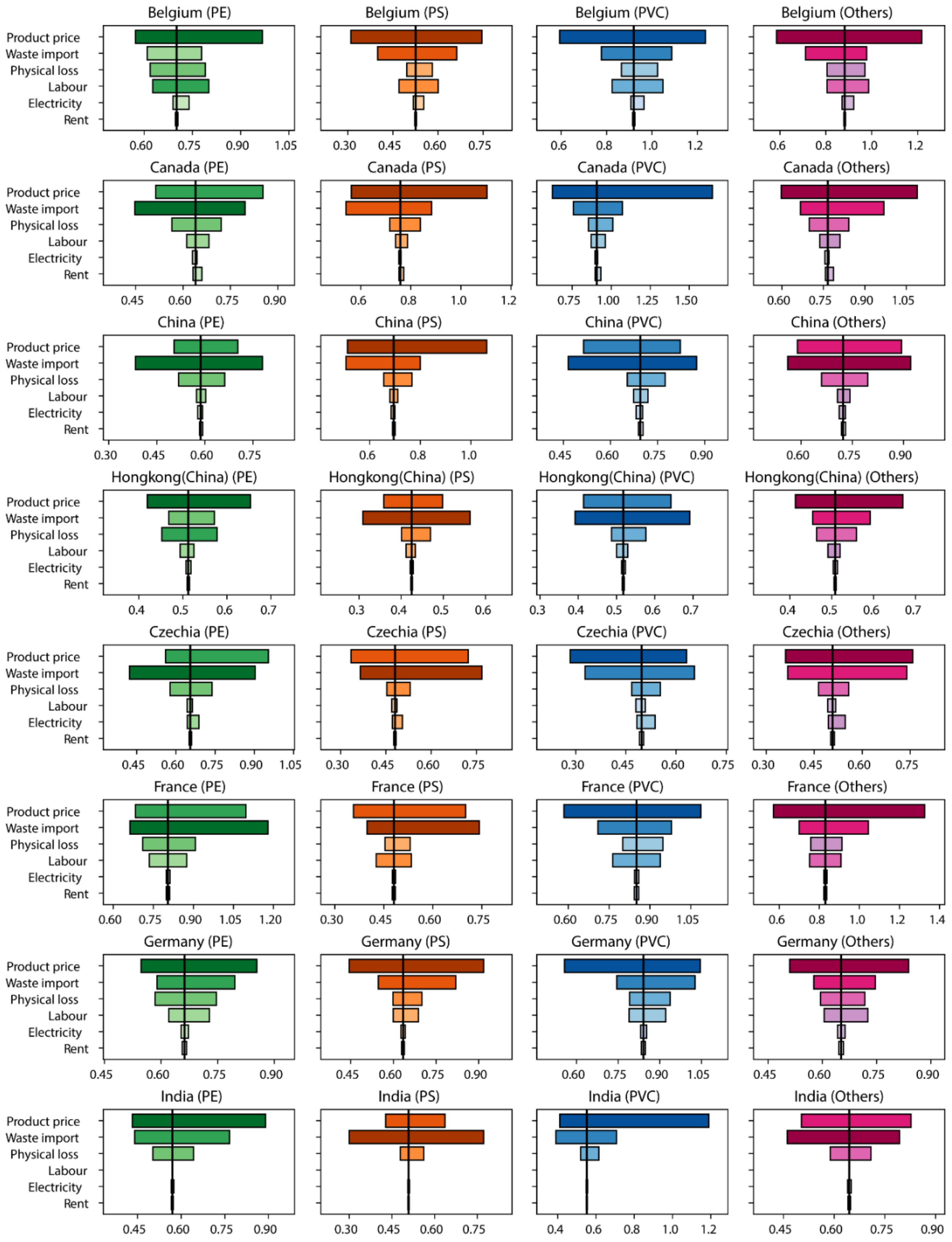

Supplementary Figure 8: Sensitivity analysis of *RRR* in 22 research countries (part 1 in 3). The *RRR* is calculated using the trade data of plastic waste imports and primary plastics exports reported by the 22 research countries. The length and colour depth of the horizontal bars are proportional to the range of sensitivity results derived from pessimistic and optimistic cases.

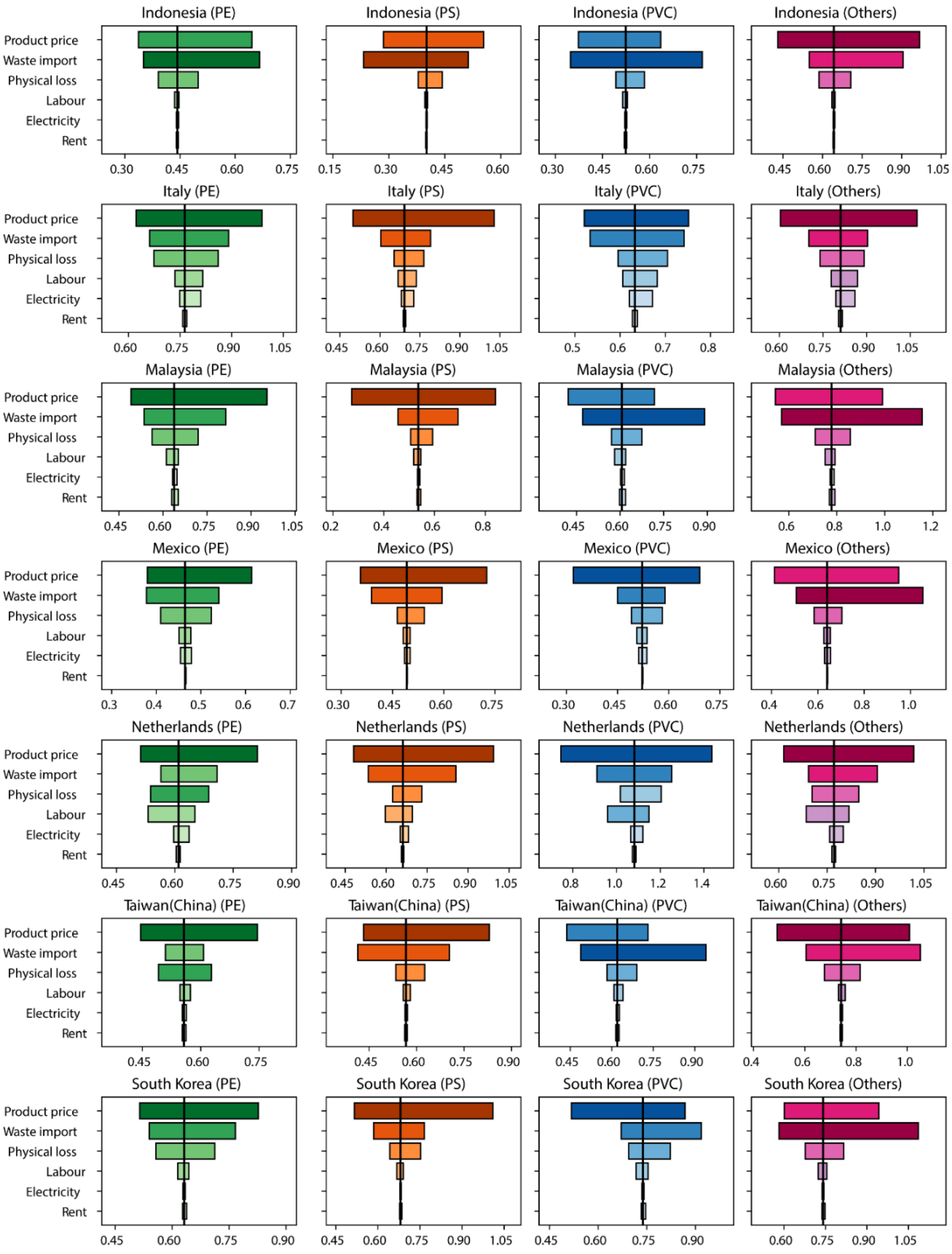

Supplementary Figure 9: Sensitivity analysis of *RRR* in 22 research countries (part 2 in 3). The *RRR* is calculated using the trade data of plastic waste imports and primary plastics exports reported by the 22 research countries. The length and colour depth of the horizontal bars are proportional to the range of sensitivity results derived from pessimistic and optimistic cases.

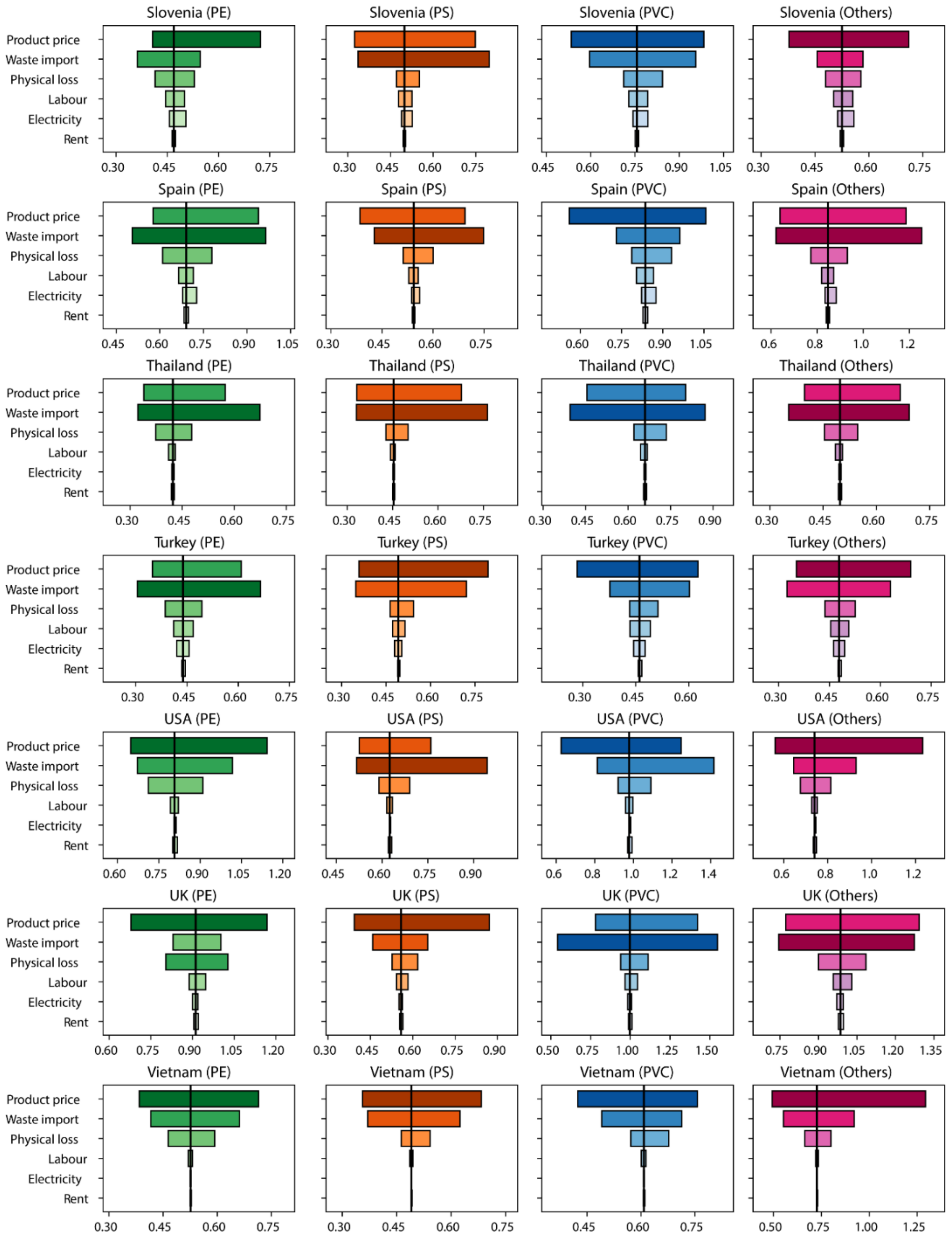

Supplementary Figure 10: Sensitivity analysis of *RRR* in 22 research countries (part 3 in 3). The *RRR* is calculated using the trade data of plastic waste imports and primary plastics exports reported by the 22 research countries. The length and colour depth of the horizontal bars are proportional to the range of sensitivity results derived from pessimistic and optimistic cases.

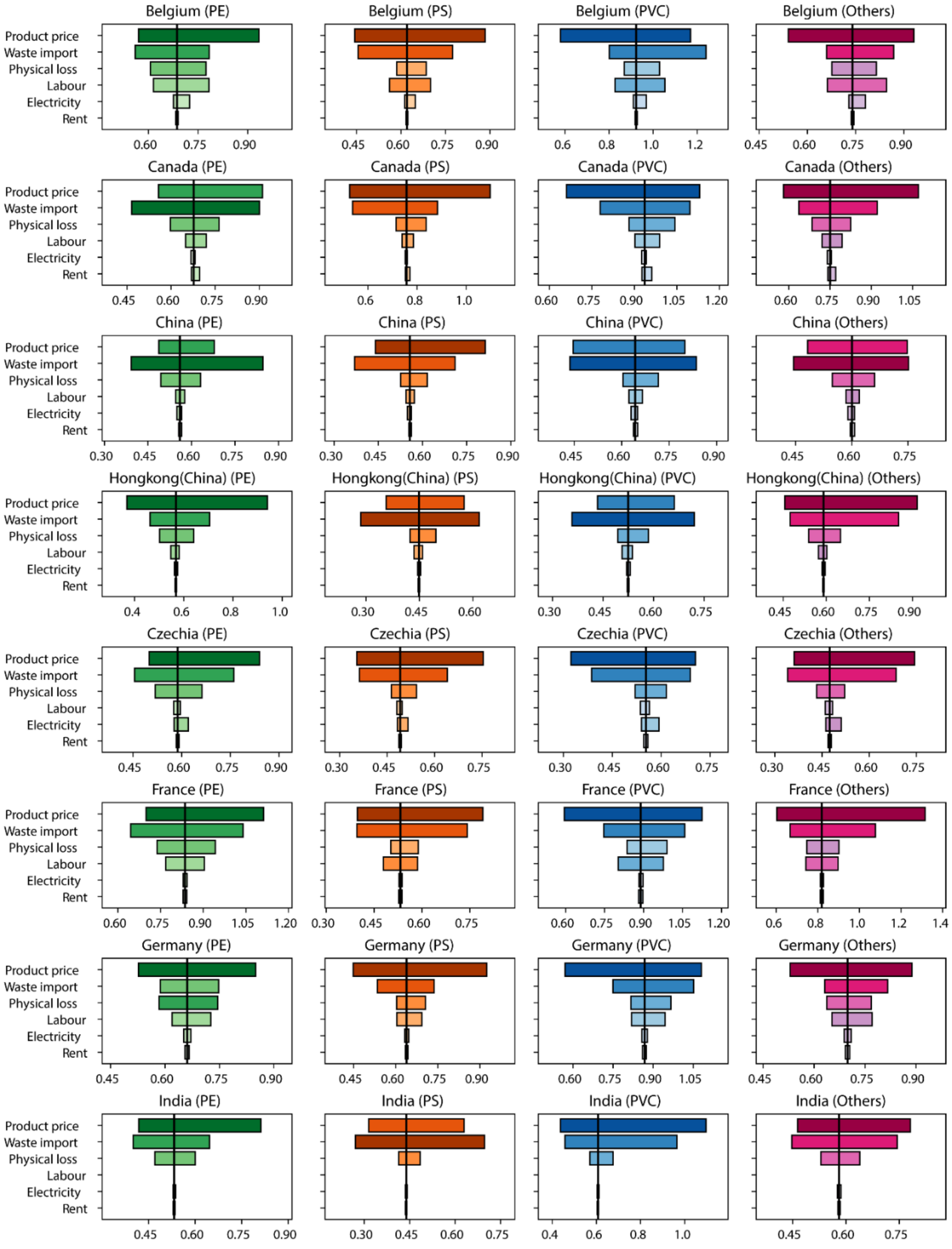

Supplementary Figure 11: Sensitivity analysis of *RRR* in 22 research countries (mirror trade data; part 1 in 3). The *RRR* is calculated using the trade data of plastic waste exports and primary plastics imports reported by the trading partners of the 22 research countries. The length and colour depth of the horizontal bars are proportional to the range of sensitivity results derived from pessimistic and optimistic cases.

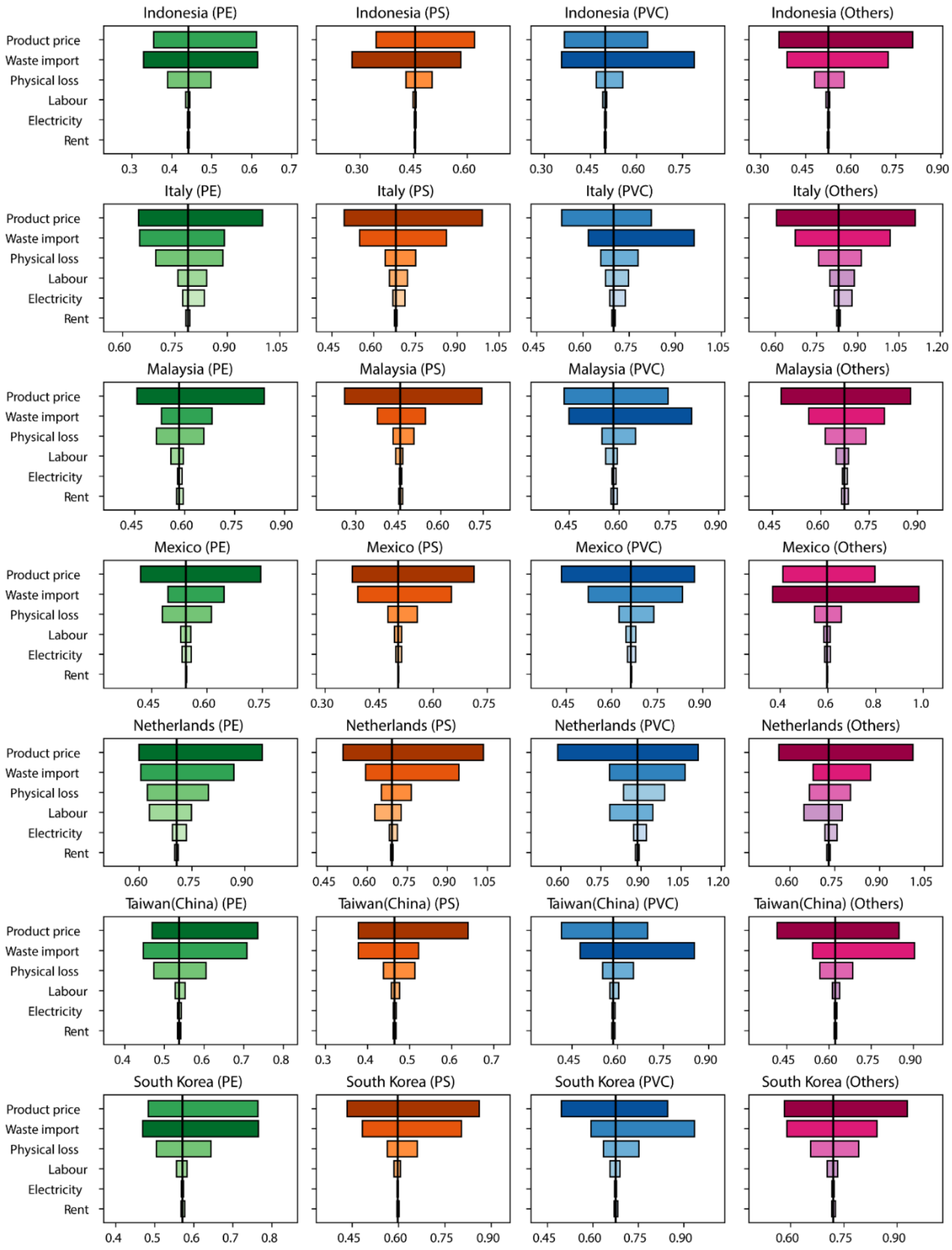

Supplementary Figure 12: Sensitivity analysis of *RRR* in 22 research countries (mirror trade data; part 2 in 3). The *RRR* is calculated using the trade data of plastic waste exports and primary plastics imports reported by the trading partners of the 22 research countries. The length and colour depth of the horizontal bars are proportional to the range of sensitivity results derived from pessimistic and optimistic cases.

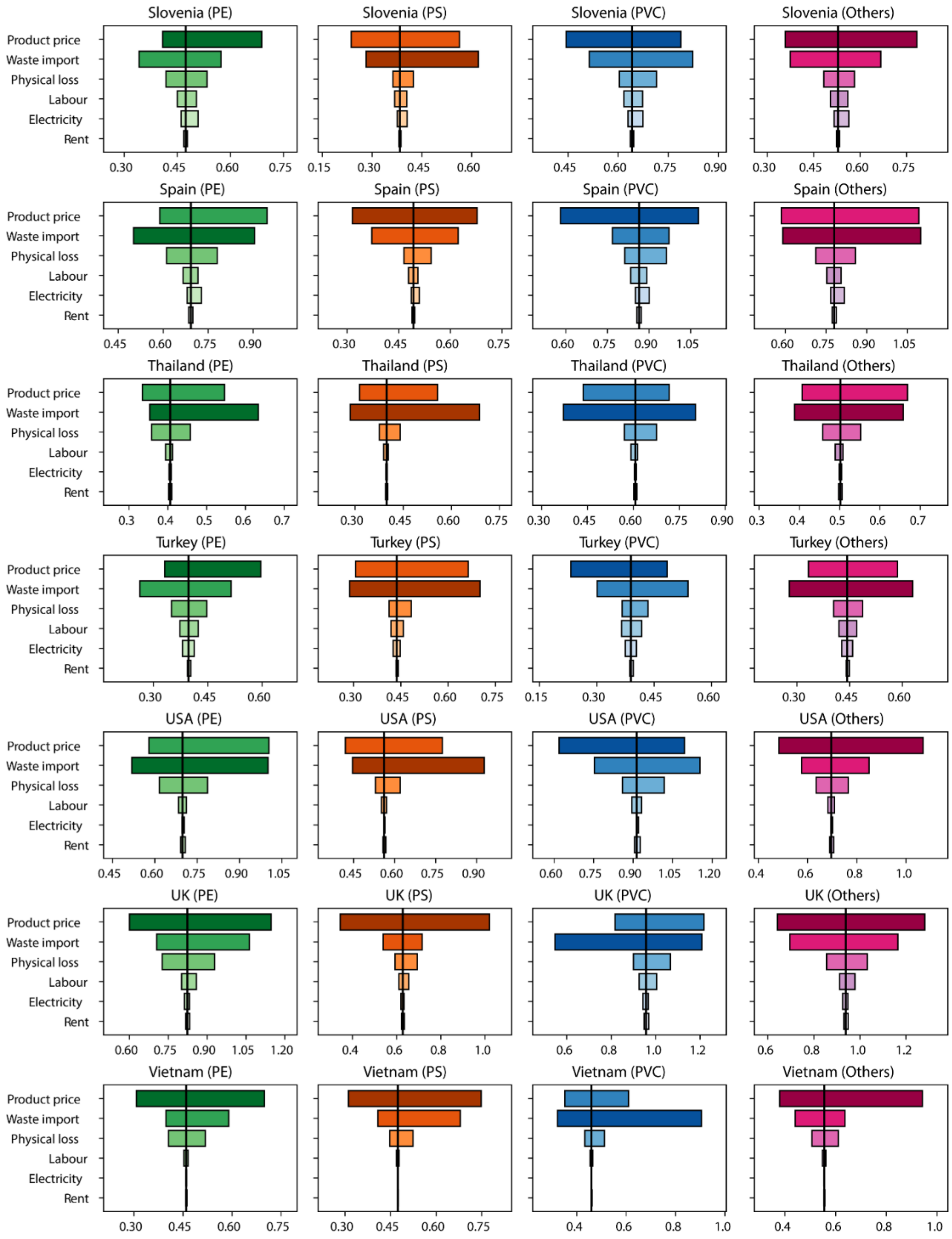

Supplementary Figure 13: Sensitivity analysis of *RRR* in 22 research countries (mirror trade data; part 3 in 3). The *RRR* is calculated using the trade data of plastic waste exports and primary plastics imports reported by the trading partners of the 22 research countries. The length and colour depth of the horizontal bars are proportional to the range of sensitivity results derived from pessimistic and optimistic cases.

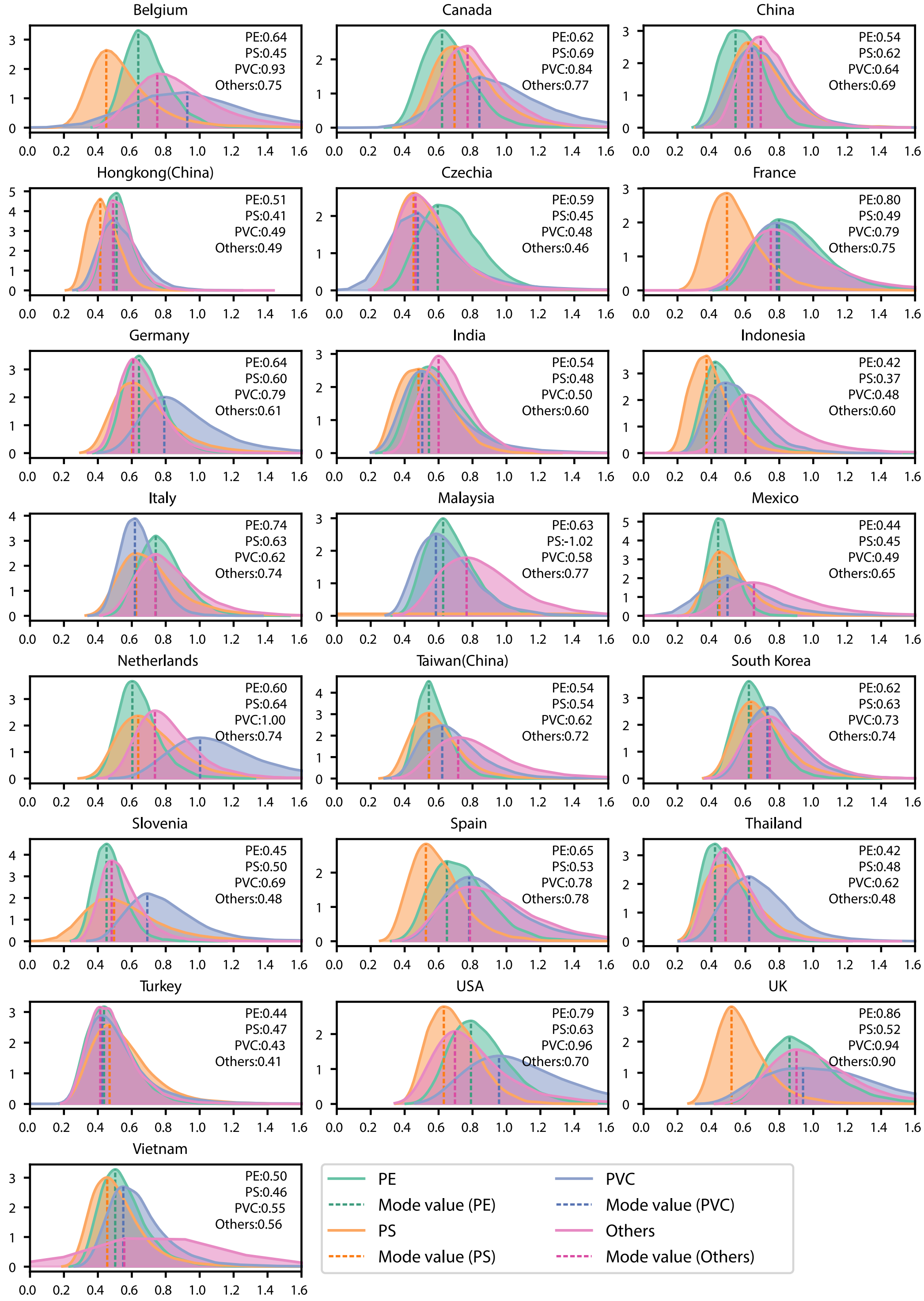

Supplementary Figure 14: Monte Carlo simulation of *RRR* in 22 research countries. The *RRR* is calculated using the trade data of plastic waste imports and primary plastics exports reported by the 22 research countries. The resulting uncertainties are propagated with a Monte Carlo simulation (sampling of 30000) using kernel density smoothing (see methods).

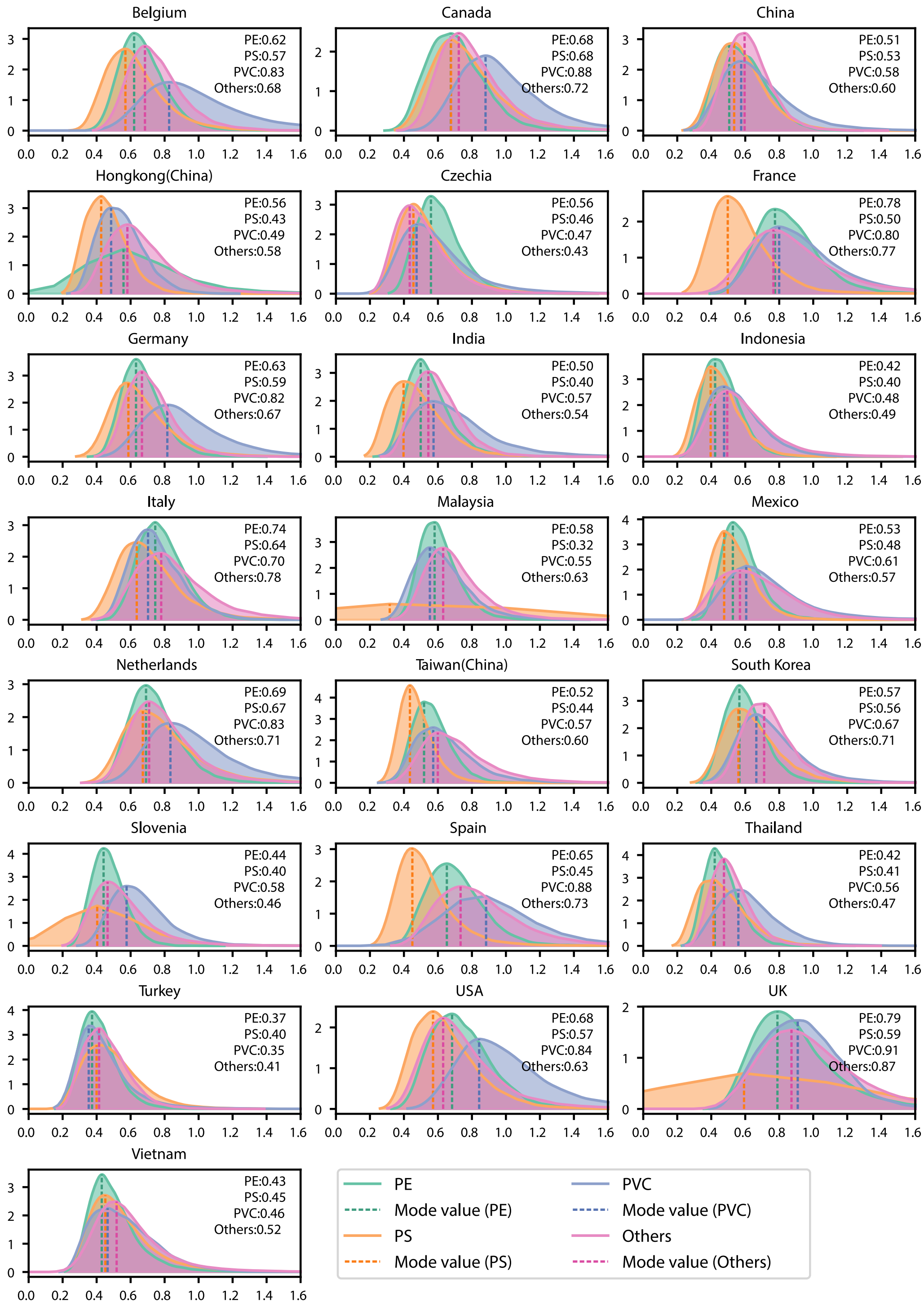

Supplementary Figure 15: Monte Carlo simulation of *RRR* in 22 research countries (mirror trade data). The *RRR* is calculated using the trade data of plastic waste exports and primary plastics imports reported by the trading partners of the 22 research countries. The resulting uncertainties are propagated with a Monte Carlo simulation (sampling of 30000) using kernel density smoothing (see methods).

3. Supplementary References

1. Gradus RHJM, Nillesen PHL, Dijkgraaf E, van Koppen RJ. A Cost-effectiveness Analysis for Incineration or Recycling of Dutch Household Plastic Waste. *Ecological Economics* **135**, 22-28 (2017).

2. Lase IS*, et al.* Method to Develop Potential Business Cases of Plastic Recycling from Urban Areas: A Case Study on Nonhousehold End-Use Plastic Film Waste in Belgium. *ACS Sustainable Chemistry & Engineering* **11**, 12677-12694 (2023).

3. Larrain M*, et al.* Techno-economic assessment of mechanical recycling of challenging post-consumer plastic packaging waste. *Resources, Conservation and Recycling* **170**, 105607 (2021).

4. Kim D-W, Kim K, Baek C-R, Phae C-G. A Study on the Calculation of the Standard Recycling Cost of PVC Profiles and Flooring Waste in Korea. *Recycling* **8**, 37 (2023).

5. Nikiema J, Asiedu Z. A review of the cost and effectiveness of solutions to address plastic pollution. *Environmental Science and Pollution Research* **29**, 24547-24573 (2022).

6. da Cruz NF, Ferreira S, Cabral M, Simões P, Marques RC. Packaging waste recycling in Europe: Is the industry paying for it? *Waste Management* **34**, 298-308 (2014).

7. Genc A, Zeydan O, Sarac S. Cost analysis of plastic solid waste recycling in an urban district in Turkey. *Waste Management & Research* **37**, 906-913 (2019).

8. *Beyond Plastics and The Last Beach CleanUp. The Real Truth About the U.S. Plastics Recycling Rate. https://bit.ly/US-plastics-recycling-rate (2022).*

9. *United States Environmental Protection Agency. Advancing Sustainable Materials Management: 2018 Tables and Figures. https://www.epa.gov/sites/default/files/2021-01/documents/2018_tables_and_figures_dec_2020_fnl_508.pdf (2020).*

10. *United States Environmental Protection Agency. National Overview: Facts and Figures on Materials, Wastes and Recycling.* https://www.epa.gov/facts-and-figures-about-materials-waste-and-recycling/national-overview-facts-and-figures-materials#:~:text=These%20Facts%20and%20Figures%20are,25%20million%20tons%20were%20composted. *(2022).*

11. *Environment and Climate Change Canada. Economic study of the Canadian plastic industry, markets and waste. https://publications.gc.ca/site/eng/9.871296/publication.html (2019).*

12. *Government of Canada. Canada one-step closer to zero plastic waste by 2030* https://www.canada.ca/en/environment-climate-change/news/2020/10/canada-one-step-closer-to-zero-plastic-waste-by-2030.html *(2020).*

13. *LACRUS. Mexico on the way to the circular economy.* https://lacrus.org/2020/08/04/mexico-on-the-way-to-the-circular-economy/ *(2020).*

14. *Government of Mexico. Urban Solid Waste (RSU).* https://www.gob.mx/semarnat/acciones-y-programas/residuos-solidos-urbanos-rsu#:~:text=Informaci%C3%B3n%20sobre%20residuos%20s%C3%B3lidos%20urbanos.&text=En%20M%C3%A9xico%20se%20generan%20diariamente,9.63%25%20de%20los%20residuos%20generados. *(2017).*

15. *Plastics Europe. The Circular Economy for Plastics – A European Overview. https://plasticseurope.org/wp-content/uploads/2022/06/PlasticsEurope-CircularityReport-2022_2804-Light.pdf (2022).*

16. *Eurostat. Municipal waste by waste management operations.* https://ec.europa.eu/eurostat/databrowser/view/env_wasmun/default/table?lang=en *(2023).*

17. Preka R, Fiorentino G, Carolis R, Barberio G. The challenge of plastics in a circular perspective. *Frontiers in Sustainable Cities* **4**, 920242 (2022).

18. *Government of the United Kingdom. OUR WASTE, OUR RESOURCES: A STRATEGY FOR ENGLAND. https://assets.publishing.service.gov.uk/government/uploads/system/uploads/attachment_data/file/765914/resources-waste-strategy-dec-2018.pdf (2018).*

19. *European Environment Agency. Municipal waste management in the Czech Republic. https://www.eea.europa.eu/publications/managing-municipal-solid-waste/czech-republic-municipal-waste-management (2013).*

20. *Environmental Protection Department. Monitoring of Solid Waste in Hong Kong Waste Statistics for 2021.* https://www.wastereduction.gov.hk/sites/default/files/msw2021.pdf *(2022).*

21. *Environmental Protection Department. Monitoring of Solid Waste in Hong Kong Waste Statistics for 2021. https://www.wastereduction.gov.hk/sites/default/files/resources_centre/waste_statistics/msw2021_eng.pdf (2022).*

22. *Environmental Protection Department of Hongkong. Hong Kong Solid Waste Monitoring Report 2021. https://www.wastereduction.gov.hk/sites/default/files/resources_centre/waste_statistics/msw2021_tc.pdf (2022).*

23. *China National Resources Recycling Association. China recycled plastics industry development report 2022. http://www.replastics.org/news_detail.php?rid=36&id=1365 (2022).*

24. *China-Italy Chamber of Commerce. THEMATIC REPORT 15 China Municipal Solid Waste Management Industry. https://www.sicab.net/wp-content/uploads/2020/05/15.-China-Municipal-Solid-Waste-Management-Industry-Report.pdf (2020).*

25. *Ministry of Ecology and Environment of the People's Republic of China. 2020 National Annual Report on the Prevention and Control of Environmental Pollution from Solid Waste in Large and Medium-sized Cities. https://www.mee.gov.cn/ywgz/gtfwyhxpgl/gtfw/202012/P020201228557295103367.pdf (2020).*

26. *Environmental Protection Administration. Solid Waste Statistics. https://www.epa.gov.tw/eng/513B0B39D090DE4C (2022).*

27. *Environmental information Center. The amount of garbage is still increasing and the most people will burn it. Two charts look at the current status of Taiwan's garbage disposal in 2022.* https://e-info.org.tw/node/237224#:~:text=%E6%A0%B9%E6%93%9A%E7%92%B0%E4%BF%9D%E7%BD%B2%E7%B5%B1%E8%A8%88%EF%BC%8C%E7%B9%BC,%E5%B9%B4%E5%8F%B0%E7%81%A3%E5%9E%83%E5%9C%BE%E8%99%95%E7%90%86%E7%8F%BE%E6%B3%81%E3%80%82 *(2023).*

28. *Ministry of Environment (Taiwan C. https://www.moenv.gov.tw/DisplayFile.aspx?FileID=DCF2E75FD363C51B&P=dc87da4b-5041-4d4c-a2f8-61c963c80aed (2023).*

29. *The Circulate Initiative. Mapping Local Plastic Recycling Supply Chains: Insights from Selected Cities in India, Indonesia, Thailand and Vietnam.* https://www.thecirculateinitiative.org/_files/ugd/77554d_9e849f45d51b4a2ebe01d12544f4a8cd.pdf?index=true *(2023).*

30. *The Economic Times. India generates 3.5 million tonnes plastic waste annually: Environment Minister.* https://economictimes.indiatimes.com/industry/environment/india-generates-3-5-million-tonnes-plastic-waste-annually-environment-minister/articleshow/90668558.cms?utm_source=contentofinterest&utm_medium=text&utm_campaign=cppst *(2022).*

31. Jang Y-C, Lee G, Kwon Y, Lim J-h, Jeong J-h. Recycling and management practices of plastic packaging waste towards a circular economy in South Korea. *Resources, Conservation and Recycling* **158**, 104798 (2020).

32. *Greenpeace. 'Single-use plastic waste' increases to the largest ever during the Corona period.* https://www.greenpeace.org/korea/press/25876/%EB%B3%B4%EB%8F%84%EC%9E%90%EB%A3%8C-%EC%BD%94%EB%A1%9C%EB%82%98-%EA%B8%B0%EA%B0%84-%EC%9D%BC%ED%9A%8C%EC%9A%A9-%ED%94%8C%EB%9D%BC%EC%8A%A4%ED%8B%B1-%ED%8F%90%EA%B8%B0%EB%AC%BC-presslease-plastic-repo/ *(2023).*

33. *Ecostar. The state of waste management in Turkey: how is it going?* https://ecostar.eu.com/state-of-plastics-waste-management-in-turkey/ *(2023).*

34. *Nicholas Institute for Environmental. Plastic Pollution Policy Country Profile: Turkey. https://nicholasinstitute.duke.edu/sites/default/files/projects/Plastic-Pollution-Policy-Country-Profile-Turkey.pdf (2022).*

35. *Minister of Environment and Water (Malaysia). Malaysia Plastics sustainability roadmap 2021-2030. https://www.kasa.gov.my/resources/alam-sekitar/MALAYSIA-PLASTICS-SUSTAINABILITY-ROADMAP-2021-2030.pdf (2021).*

36. Sulaiman SA, Ahmad RK. Plastic Waste Issue in Malaysia: Where Are We? In: *Energy and Environment in the Tropics* (ed Sulaiman SA). Springer Nature Singapore (2023).

37. *World Economic Forum. Radically reducing plastic pollution in Indonesia: A multistakeholder action plan national plastic action partnership. https://pacecircular.org/sites/default/files/2021-03/NPAP-Indonesia-Multistakeholder-Action-Plan_April-2020_compressed%20%281%29.pdf (2020).*

38. *ZeroWaste Center. Plastics Waste Facts in Indonesia.* https://zerowastecenter.org/plastics-waste-facts-in-indonesia/17678/ *(2021).*

39. *World Bank Group. Plastic Waste Material Flow Analysis for Thailand – Summary Report. http://hdl.handle.net/10986/37182 (2022).*

40. *SAWASDEE Thailand. Statistics of Plastic Waste in Thailand That Can Be Solved By All Sectors.* https://thailand.go.th/issue-focus-detail/001_04_028 *(2023).*

41. *IUCN-EA-QUANTIS. National Guidance for plastic pollution hotspotting and shaping action for Vietnam.* https://www.iucn.org/sites/default/files/content/documents/2021/vietnam_-_national_guidance_for_plastic_pollution_hotspotting_and_shaping_action.pdf *(2020).*

42. *Ministry of Natural Resources and Environment (MONRE). National State of Environment Report 2019 on Solid Waste. https://www.vd-office.org/en/national-state-of-environment-report-2019-on-solid-waste-released/#:~:text=The%20daily%20waste%20generation%20in,area%20generated%2028.394%20tons%2Fday. (2020).*

43. *Yen NT. Plastic waste management in Viet Nam. https://www.env.go.jp/en/recycle/asian_net/Annual_Workshops/2019_PDF/Session1/S1_12_Vietnam_ANWS2019.pdf (2019).*

44. *UNCTAD. Classifications.* https://unctadstat.unctad.org/EN/Classifications.html *(2023).*

45. *World Bank Group. Consumer price index (2010 = 100) - United States.* https://data.worldbank.org/indicator/FP.CPI.TOTL?end=1983&locations=US (Accessed 05/24/2024) *(2024).*

46. Chiong MS, Chun Y-Y, Tsukahara K, Tahara K. An Analysis of Practices and Challenges for Plastic Recycling Industry in Malaysia. *International Journal of Automation Technology* 16, (2022).

47. *WWF Thailand. Scaling up circular strategies to achieve zero plastic waste in Thailand.* https://wwfint.awsassets.panda.org/downloads/zero_plastic_waste_in_thailand_en.pdf *(2020).*

48. *WFF-Philippines. EPR scheme assessment for plastic packing waste in the Philippines.* https://wwf.org.ph/wp-content/uploads/2020/12/WWF_REPORT_EPR_Philippines_2020.pdf *(2020).*

49. Hossain MU, Ng ST, Dong Y, Amor B. Strategies for mitigating plastic wastes management problem: A lifecycle assessment study in Hong Kong. *Waste Management* **131**, 412-422 (2021).

50. Lai Y-Y, Lee Y-M. Management strategy of plastic wastes in Taiwan. *Sustainable Environment Research* **32**, 11 (2022).

51. *Japan Plastic Waste Management Institute. An Introduction to Plastic Recycling in Japan 2022. https://www.pwmi.or.jp/ei/plastic_recycling_2022.pdf (2022).*

52. Drewniok MP, Gao Y, Cullen JM, Cabrera Serrenho A. What to Do about Plastics? Lessons from a Study of United Kingdom Plastics Flows. *Environmental Science & Technology* **57**, 4513-4521 (2023).

53. Basuhi R, Moore E, Gregory J, Kirchain R, Gesing A, Olivetti EA. Environmental and economic implications of U.S. postconsumer plastic waste management. *Resources, Conservation and Recycling* **167**, 105391 (2021).

54. Sevigné-Itoiz E, Gasol CM, Rieradevall J, Gabarrell X. Contribution of plastic waste recovery to greenhouse gas (GHG) savings in Spain. *Waste Management* **46**, 557-567 (2015).

55. Picuno C, Alassali A, Chong ZK, Kuchta K. Flows of post-consumer plastic packaging in Germany: An MFA-aided case study. *Resources, Conservation and Recycling* **169**, 105515 (2021).

56. *Amadei A, Ardente F. Modelling plastic flows in the European Union value chain. https://publications.jrc.ec.europa.eu/repository/bitstream/JRC130613/JRC130613_01.pdf (2022).*

57. *Chatham House. 'resourcetrade.earth'.* https://resourcetrade.earth/ *(2024).*

58. *Plastic Recyclers Europe. Plastic recycling industry figures 2022: Mapping of installed capacities. https://www.plasticsrecyclers.eu/wp-content/uploads/2024/01/Plastics-Recycling-Industry-in-Europe-2022-data.pdf (2022).*

59. *American Chemistry Council. Economic impact of advanced plastics recycling and recovery facilities in the US. https://www.americanchemistry.com/content/download/10845/file/Potential-Economic-Impact-of-Advanced-Recycling-Recovery-Facilities-in-the-US.pdf (2019).*

60. *United States Environmental Protection Agency. 2020 Recycling Economic Information (REI) Report. https://www.epa.gov/sites/default/files/2020-11/documents/rei_report_508_compliant.pdf (2020).*

61. *ALPLA. ALPLA puts HDPE recycling plant in Mexico into operation.* https://blog.alpla.com/en/press-release/newsroom/alpla-puts-hdpe-recycling-plant-mexico-operation/11-21 *(2021).*

62. *China Plastic Recycling Association. Survey report on employees in the waste plastic recycling industry. http://www.replastics.org/news_detail.php?rid=44&cid=0&id=1034 (2022).*

63. Shanker R*, et al.* Plastic waste recycling: existing Indian scenario and future opportunities. *International Journal of Environmental Science and Technology* **20**, 5895-5912 (2023).

64. *Indorama Polyester Industries Public Company Limited. Indorama Polyester Industries Public Company Limited – Nakhon Pathom, Thailand.* https://www.indoramaventures.com/en/worldwide/815/indorama-polyester-industries-nakhon-pathom *(2023).*

65. *USDA National Agricultural Library. LCA Commons.* https://doi.org/10.15482/USDA.ADC/1173236 *(2015).*

66. Wernet G, Bauer C, Steubing B, Reinhard J, Moreno-Ruiz E, Weidema B. The ecoinvent database version 3 (part I): overview and methodology. *The International Journal of Life Cycle Assessment* **21**, 1218-1230 (2016).

67. Lim J, Ahn Y, Kim J. Optimal sorting and recycling of plastic waste as a renewable energy resource considering economic feasibility and environmental pollution. *Process Safety and Environmental Protection* **169**, 685-696 (2023).

68. Shan C, Pandyaswargo AH, Onoda H. Environmental Impact of Plastic Recycling in Terms of Energy Consumption: A Comparison of Mechanical and Chemical Recycling Technologies. *Energies* **16**, 2199 (2023).

69. Civancik-Uslu D*, et al.* Moving from linear to circular household plastic packaging in Belgium: Prospective life cycle assessment of mechanical and thermochemical recycling. *Resources, Conservation and Recycling* **171**, 105633 (2021).

70. Schwarz AE, Ligthart TN, Godoi Bizarro D, De Wild P, Vreugdenhil B, van Harmelen T. Plastic recycling in a circular economy; determining environmental performance through an LCA matrix model approach. *Waste Management* **121**, 331-342 (2021).

71. Meys R, Frick F, Westhues S, Sternberg A, Klankermayer J, Bardow A. Towards a circular economy for plastic packaging wastes – the environmental potential of chemical recycling. *Resources, Conservation and Recycling* **162**, 105010 (2020).

72. Uekert T*, et al.* Technical, economic, and environmental comparison of closed-loop recycling technologies for common plastics. *ACS Sustainable Chemistry & Engineering* **11**, 965-978 (2023).

73. *Tenaga Nasional Berhad. Electricity tariff schedule.* https://www.tnb.com.my/assets/files/Tariff_Rate_Final_1.June.2011.pdf *(2011).*

74. *Baker McKenzie. Indonesian Government publishes 2017 Cost of Generation (BPP) figures.* https://www.bakermckenzie.com/-/media/files/insight/publications/2018/04/al_jakarta_indonesiangovcostofgeneration_apr18.pdf?la=en *(2018).*

75. *BAKER McKenzie. Indonesia: Government publishes PLN's 2020 Cost of Generation (BPP) figures.* https://insightplus.bakermckenzie.com/bm/projects/indonesia-government-publishes-plns-2020-cost-of-generation-bpp-figures *(2021).*

76. *Prime Minister of Vietnam. Decision No. 268/QD-TTg providing the electricity retail price tariff.* https://vanbanphapluat.co/decision-no-268-qd-ttg-providing-the-electricity-retail-price-tariff *(2011).*

77. *Prime Minister of Vietnam. Decision No. 28/2014/QD-TTg regulations on structure of electricity retail tariff.* https://vanbanphapluat.co/decision-no-28-2014-qd-ttg-regulations-on-structure-of-electricity-retail-tariff *(2014).*

78. *Ministry of Industry and Trade (Vietnam). Decision on adjustment of average electricity retail price and regulations on electricity price.* https://www.idico.com.vn/vnt_upload/project/02_2020/Decision_No._648.QD-BCT.pdf *(2019).*

79. *Provincial Electricity Authority (Thailand). Electricity Tariffs.* https://www.pea.co.th/Portals/_default/Documents/Rate2015.pdf *(2015).*

80. *Provincial Electricity Authority (Thailand). Electricity Tariffs.* https://www.pea.co.th/Portals/1/demand_response/Electricity%20Tariffs%20Nov61.pdf?ver=2018-11-21-145427-433 *(2018).*

81. *Delhi electricity regulatory commission. Tariff schedule NDMC for FY 2014-15.* https://www.ndmc.gov.in/public_notice/TARIFF_SCHEDULE.pdf *(2014).*

82. *Delhi electricity regulatory commission. Tariff schedule for FY 2015-16.* https://www.derc.gov.in/tariff-order-fy-2015-16 *(2015).*

83. *Delhi electricity regulatory commission. Tariff schedule for FY 2017-18.* https://www.bsesdelhi.com/documents/73527/74654/tariff+schedule+.pdf/393725eb-ada4-6370-7811-0eeb1bc39573?t=1517809125427 *(2017).*

84. *Delhi electricity regulatory commission. Tariff schedule for FY 2018-19.* https://tatapower-ddl.com/Editor_UploadedDocuments/Content/Electricity%20Tariff%20schedule%20for%20FY%2018-19.pdf *(2018).*

85. *Delhi electricity regulatory commission. Tariff schedule for FY 2019-20.* https://www.bsesdelhi.com/documents/73527/74654/TARIFF_SCHEDULE_FY_2019_20.pdf/a26ae227-12d7-e361-7a3a-1ef753f58b16?t=1614774782392 *(2019).*

86. *Delhi electricity regulatory commission. Tariff schedule for FY 2020-21.* https://www.derc.gov.in/sites/default/files/Tariff%20Schedule%202020-21.pdf *(2020).*

87. *Delhi electricity regulatory commission. Tariff schedule for FY 2021-22.* https://tatapower-ddl.com/Editor_UploadedDocuments/Content/Press_Release_2022_Eng_30.09.2021.pdf *(2021).*

88. *National Energy Administration (China). National Electricity Price Regulatory Notice 2013-2014.* http://zfxxgk.nea.gov.cn/auto92/201509/t20150902_1959.htm *(2015).*

89. *National Energy Administration (China). National Electricity Price Regulatory Notice 2015.* http://zfxxgk.nea.gov.cn/auto92/201611/t20161101_2312.htm *(2016).*

90. *National Energy Administration (China). National Electricity Price Regulatory Notice 2016.* https://www.gov.cn/xinwen/2017-12/31/5252010/files/a864b93b07da422f9a3998750ba1c3d3.pdf *(2017).*

91. *National Energy Administration (China). National Electricity Price Regulatory Notice 2017.* https://www.gov.cn/xinwen/2018-10/09/5328808/files/fe3fe19e7cc646b39338fdab3e24fb3a.pdf *(2018).*

92. *National Energy Administration (China). National Electricity Price Regulatory Notice 2018.* http://www.nea.gov.cn/138530255_15729388881531n.pdf *(2018).*

93. *National Energy Administration (China). The development of China's electricity market in 2022.* https://www.ndrc.gov.cn/fggz/hjyzy/jnhnx/202302/t20230215_1348801.html *(2023).*

94. *CLP Power Hong Kong Limited. CLP announces 2013 tariff adjustment and introduce energy saving rebate.* https://www.clpgroup.com/content/dam/clp-group/channels/media/document/2012/20121211_Eng.pdf.coredownload.pdf *(2012).*

95. *Limited CPHK. CLP announced the adjustment of electricity price in 2018.* https://www.clpgroup.com/content/dam/clp-group/channels/media/document/2017/20171212_tc.pdf.coredownload.pdf *(2017).*

96. *CLP Power Hong Kong Limited. CLP electricity tariff in 2021.* https://www.clp.com.hk/content/dam/clphk/documents/customer-service-site/tariff-site/Tariff%20Table-Chinese%20(2021-01-01).pdf *(2021).*

97. *Taiwan Business TOPICS. The High Cost of Taiwan's Low Electricity Prices.* https://topics.amcham.com.tw/2022/05/the-high-cost-of-taiwans-low-electricity-prices/ *(2022).*

98. *S&P Global Commodity Insights. S Korea raises electricity rate for first time in around 8 years on fuel costs.* https://www.spglobal.com/commodityinsights/en/market-insights/latest-news/coal/092321-s-korea-raises-electricity-rate-for-first-time-in-around-8-years-on-fuel-costs *(2021).*

99. *Korea Electric Power Corporation. Electric Rates Table.* https://home.kepco.co.kr/kepco/EN/F/htmlView/ENFBHP00103.do?menuCd=EN060201 *(2023).*

100. *Turkish Statistical Institute. Electricity and Natural Gas Prices, I. Period: January–June, 2013.* https://data.tuik.gov.tr/Bulten/Index?p=electricity-and-natural-gas-prices-i.-period:-january-june,-2013-15883&dil=2 *(2013).*

101. *Turkish Statistical Institute. Electricity and Natural Gas Prices, II. Period: July-December, 2013.* https://data.tuik.gov.tr/Bulten/Index?p=electricity-and-natural-gas-prices-ii.-period:-july-december,-2013-15921&dil=2 *(2014).*

102. *Turkish Statistical Institute. Electricity and Natural Gas Prices, I. Period: January–June, 2014.* https://data.tuik.gov.tr/Bulten/Index?p=electricity-and-natural-gas-prices-i.-period:-january-june,-2014-15922&dil=2 *(2014).*

103. *Turkish Statistical Institute. Electricity and Natural Gas Prices, II. Period: July-December, 2014.* https://data.tuik.gov.tr/Bulten/Index?p=electricity-and-natural-gas-prices-ii.-period:-july-december,-2014-18758&dil=2 *(2015).*

104. *Turkish Statistical Institute. Electricity and Natural Gas Prices, I. Period: January–June, 2015.* https://data.tuik.gov.tr/Bulten/Index?p=electricity-and-natural-gas-prices-i.-period:-january-june,-2015-18759&dil=2 *(2015).*

105. *Turkish Statistical Institute. Electricity and Natural Gas Prices, II. Period: July-December, 2015.* https://data.tuik.gov.tr/Bulten/Index?p=electricity-and-natural-gas-prices-ii.-period:-july-december,-2015-21585&dil=2 *(2016).*

106. *Turkish Statistical Institute. Electricity and Natural Gas Prices, I. Period: January–June, 2016.* https://data.tuik.gov.tr/Bulten/Index?p=electricity-and-natural-gas-prices-i.-period:-january-june,-2016-21586&dil=2 *(2016).*

107. *Turkish Statistical Institute. Electricity and Natural Gas Prices, II. Period: July-December, 2016.* https://data.tuik.gov.tr/Bulten/Index?p=electricity-and-natural-gas-prices-ii.-period:-july-december,-2016-24636&dil=2 *(2017).*

108. *Turkish Statistical Institute. Electricity and Natural Gas Prices, I. Period: January–June, 2017.* https://data.tuik.gov.tr/Bulten/Index?p=electricity-and-natural-gas-prices-i.-period:-january---june,-2017-24637&dil=2 *(2017).*

109. *Turkish Statistical Institute. Electricity and Natural Gas Prices, Period II: July–December, 2017.* https://data.tuik.gov.tr/Bulten/Index?p=electricity-and-natural-gas-prices-period-ii:-july-december,-2017-27665&dil=2 *(2018).*

110. *Turkish Statistical Institute. Electricity and Natural Gas Prices, Period I: January-June, 2018.* https://data.tuik.gov.tr/Bulten/Index?p=electricity-and-natural-gas-prices-period-i:-january-june,-2018-27666&dil=2 *(2018).*

111. *Turkish Statistical Institute. Electricity and Natural Gas Prices, Period II: July–December, 2018.* https://data.tuik.gov.tr/Bulten/Index?p=electricity-and-natural-gas-prices-period-ii:-july-december,-2018-30608&dil=2 *(2019).*

112. *Turkish Statistical Institute. Electricity and Natural Gas Prices, Period I: January-June, 2019.* https://data.tuik.gov.tr/Bulten/Index?p=electricity-and-natural-gas-prices-period-i:-january-june,-2019-30609&dil=2 *(2019).*

113. *Turkish Statistical Institute. Electricity and Natural Gas Prices, Period II: July–December, 2019.* https://data.tuik.gov.tr/Bulten/Index?p=electricity-and-natural-gas-prices-period-ii:-july-december,-2019-33646&dil=2 *(2020).*

114. *Turkish Statistical Institute. Electricity and Natural Gas Prices, Period I: January-June, 2020.* https://data.tuik.gov.tr/Bulten/Index?p=electricity-and-natural-gas-prices-period-i:-january-june,-2020-33647&dil=2 *(2020).*

115. *Turkish Statistical Institute. Electricity and Natural Gas Prices, Period II: July–December, 2020.* https://data.tuik.gov.tr/Bulten/Index?p=electricity-and-natural-gas-prices-period-ii:-july-december,-2020-37458&dil=2 *(2021).*

116. *Turkish Statistical Institute. Electricity and Natural Gas Prices, Period I: January-June, 2021.* https://data.tuik.gov.tr/Bulten/Index?p=electricity-and-natural-gas-prices-period-i:-january-june,-2021-37459&dil=2 *(2021).*

117. *Turkish Statistical Institute. Electricity and Natural Gas Prices, Period II: July–December, 2021.* https://data.tuik.gov.tr/Bulten/Index?p=electricity-and-natural-gas-prices-period-ii:-july-december,-2021-45566&dil=2 *(2022).*

118. *Turkish Statistical Institute. Electricity and Natural Gas Prices, Period I: January-June, 2022.* https://data.tuik.gov.tr/Bulten/Index?p=Electricity-and-Natural-Gas-Prices-Period-I:-January-June,-2022-45567&dil=2 *(2022).*

119. *IEA. Industrial electricity prices in India and selected countries, 2005–2019.* https://www.iea.org/data-and-statistics/charts/industrial-electricity-prices-in-india-and-selected-countries-2005-2019 *(2020).*

120. *NERA Economic Consulting. Mexican Electricity Wholesale Market Report 2019.* https://www.nera.com/content/dam/nera/publications/2020/PUB_Mexican-Electricity-Wholesale-Market-Report_013120.pdf *(2020).*

121. *Antuko. Capacity Balance Market: A plunge to zero in 2020?* https://antuko.com/capacity-balance-market-a-plunge-to-zero-in-2020/ *(2021).*

122. *Google Earth Help. Measure distances and areas in Google Earth.* https://support.google.com/earth/answer/9010337?hl=en&co=GENIE.Platform%3DDesktop *(2023).*

123. *Thailand board of investment. Costs of doing business in Thailand 2014. https://image.mfa.go.th/mfa/0/fJHW5PV5Yy/migrate_directory/business-20150713-113434-334538.pdf (2014).*

124. *Thailand board of investment. Costs of doing business in Thailand 2016. https://aseanup.com/guide-doing-business-thailand/ (2016).*

125. *Thailand board of investment. Costs of doing business in Thailand 2017. https://www.slideshare.net/boinyc/costs-of-doing-business-in-thailand-2017 (2017).*

126. *Thailand board of investment. Costs of doing business in Thailand 2018. https://www.slideshare.net/boinyc/costs-of-doing-business-in-thailand-2018 (2018).*

127. *Thailand board of investment. Costs of doing business in Thailand 2019. https://www.boi.go.th/upload/content/Cost%20of%20Doing%20Business%202019_Online_5c6a5d3a3c43b.pdf (2019).*

128. *Thailand board of investment. Costs of doing business in Thailand 2020. https://www.boi.go.th/upload/content/Cost_of_Doing_Business2020.pdf (2020).*

129. *Thailand board of investment. Costs of doing business in Thailand 2022. https://www.slideshare.net/boinyc/costs-of-doing-business-in-thailand-2022 (2022).*

130. *Thailand board of investment. Costs of doing business in Thailand 2023. https://www.boi.go.th/upload/content/Cost_of_Doing_Business.pdf (2023).*

131. *Cushman & Wakefield. Marketbeat: Greater Jakarta Industrial Q3 2020. https://www.cushmanwakefield.com/en/thailand/insights/thailand-marketbeat (2020).*

132. *Cushman & Wakefield. Marketbeat: Kuala Lumpur Industrial Q2 2021. https://cw-gbl-gws-prod.azureedge.net/-/media/cw/marketbeat-pdfs/2021/q2/apac-and-gc/malaysia--kuala-lumpur--industrial-q2-2021.pdf?rev=f248c82c4ea1468b9fd82bf26e8eb343 (2021).*

133. *Kizuna. Factory for rent in Vietnam: Prices, risks and solution, how to choose.* https://www.kizuna.vn/en/news/ways-to-avoid-risks-of-choosing-a-factory-for-rent-near-hcmc-629 *(2013).*

134. *Cushman & Wakefield. Marketbeat: Delhi NCR Industrial 2018. https://www.cushmanwakefield.com/-/media/cw/marketbeat-pdfs/2018/q2/india-delhi-ncr-industrial-h1-2018.pdf (2018).*

135. *Cushman & Wakefield. Marketbeat: Delhi NCR Industrial H2 2019. https://www.cushmanwakefield.com/-/media/cw/marketbeat-pdfs/2019/q4/q4-2019-apac-marketbeat-reports/india--delhi-ncr--industrial-h2-2019.pdf (2019).*

136. *Cushman & Wakefield. Marketbeat: Delhi NCR Industrial H2 2021. https://cw-gbl-gws-prod.azureedge.net/-/media/cw/marketbeat-pdfs/2021/q4/apac-and-gc/india-delhi-ncr-industrial-mb-h2-2021-final.pdf?rev=ec1a6a4694a54f97b3fb17e2f2ea5200 (2021).*

137. *Cushman & Wakefield. Marketbeat: Delhi NCR Industrial H2 2022. https://cw-gbl-gws-prod.azureedge.net/-/media/cw/marketbeat-pdfs/2022/q4/apac-and-gc/india---delhi-ncr---industrial-mb_h2-2022.pdf?rev=82d8e23f97864a67820b5bd6b830186f (2022).*

138. *Knight Frank. Shanghai Industrial Market Report 2020.* https://content.knightfrank.com/research/1591/documents/en/shanghai-industrial-market-report-q1-2020-7238.pdf *(2020).*

139. *Savills. China Industrial Real Estate 2022.* https://pdf.savills.asia/selected-international-research/221110-china-industrial-public-cn.pdf *(2022).*

140. *CBRE Global Research and Consulting. Hongkong Industrial MarketView Q2 2013.* https://f.tlcollect.com/fr2/413/40297/HK_Industrial_Q2_2013.pdf *(2013).*

141. *Knight Frank. Hong Kong Industrial Summary Q4 2021. https://content.knightfrank.com/research/2415/documents/en/hong-kong-industrial-summary-q4-2021-8879.pdf (2021).*

142. *Taiwan Industrial Land Supply and Service Information Network. Taiwan industrial rent statistics 2023.* https://idbpark.moeaidb.gov.tw/RentalReport/price_industrial *(2023).*

143. *DWS Group. South Korea real estate market annual report. https://www.dws.com/AssetDownload/Index?assetGuid=f97b7a6d-d098-4826-96d7-ad059e5699b4&consumer=E-Library (2020).*

144. *Real Estate Asia. Seoul industrial property rents slip 0.1% in 3Q21.* https://realestateasia.com/industrial/news/seoul-industrial-property-rents-slip-01-in-3q21 *(2022).*

145. *Cushman & Wakefield. Marketbeat: Turkey Industrial H2 2021. https://cw-gbl-gws-prod.azureedge.net/-/media/cw/marketbeat-pdfs/2021/q4/emea-reports/turkey-industrial-marketbeat-q4-2021.pdf?rev=9950984da3a84230b3bb941374373d29 (2021).*

146. *Cushman & Wakefield. Marketbeat: Turkey Industrial H2 2022. https://cw-gbl-gws-prod.azureedge.net/-/media/cw/marketbeat-pdfs/2022/q2/emea/turkey-industrial-marketbeat-q2-2022.pdf?rev=40e0587d6cab4fbb9390f67d62ec2866 (2022).*

147. *Cushman & Wakefield. Marketbeat: US Industrial Q2 2018. https://assets.recenter.tamu.edu/documents/mktresearch/US_Industrial_CushmanWakefield.pdf (2019).*

148. *Cushman & Wakefield. Marketbeat: US National Industrial Q4 2022. https://cw-gbl-gws-prod.azureedge.net/-/media/cw/americas/united-states/insights/marketbeats-images/us-*

marketbeats/industrial/2023/us_industrial_marketbeat_q4_2022-new.pdf?rev=428a6541d93f453cbb682ce4ae76f55e (2022).

149. *CBRE Global Research and Consulting. Canada Industrial Figures Q4 2022.* https://www.cbre.ca/insights/figures/canada-industrial-figures-q4-2022#:~:text=Market%20conditions%20remain%20tight%20across,%25%20year%2Dover%2Dyear. *(2020).*

150. *Cushman & Wakefield. Marketbeat: Monterrey Q4 2019. https://www.cushmanwakefield.com/-/media/cw/marketbeat-pdfs/2019/q4/monterrey_americas_marketbeat_industrial_q42019.pdf (2020).*

151. *Cushman & Wakefield. Marketbeat: Monterrey Q1 2022. https://cw-gbl-gws-prod.azureedge.net/-/media/cw/marketbeat-pdfs/2022/q1/latam/monterrey_americas_marketbeat_industrial_q1-2022.pdf?rev=3bd1aa45951a453e8241e3a036743d49 (2022).*

152. *Colliers International. Germany: Industrial & Logistics Market Report 2020. https://www.colliers.com/download-article?itemId=3ab20255-4ca7-4c62-a21c-f230a9eef41c (2020).*

153. *Jones Lang LaSalle. Logistics and Industrial Market Overview Germany - H1 2023. https://www.jll.de/en/trends-and-insights/research/logistics-and-Industrial-market-overview (2023).*

154. *NAI Netherlands. The State of Affairs Commercial Property 2020. https://nainetherlands.nl/wp-content/uploads/pdf/State%20of%20affairs%20-%20The%20Dutch%20commercial%20property%20market%202020.pdf (2021).*

155. *Europe Real Estate. Slovenia: city profile.* https://europe-re.com/ljubljana-colliers/50103 *(2015).*

156. *RE/MAX Commercial. Slovenian Commercial Real Estate Market Review 2020. https://www.remax-israel.com/Sites/REMAXSlovenia/RegionalWeb/porocila/REPORT_2020.pdf (2021).*

157. *Colliers International. Market Overview Slovenia H1 2021. https://www.colliers.com/en-hr/research/market-overview-slovenia-h1-2021 (2021).*

158. *Colliers International. Market Snapshot Slovenia H1 2022. https://www.colliers.com/en-si/research/market-snapshot-h1-2022-slovenia (2022).*

159. *Knight Frank. UK Logistics Market Dashboard - May 2021. https://content.knightfrank.com/research/2072/documents/en/uk-logistics-market-dashboard-may-2021-8156.pdf (2021).*

160. Jin H, Gonzalez-Gutierrez J, Oblak P, Zupančič B, Emri I. The effect of extensive mechanical recycling on the properties of low density polyethylene. *Polymer Degradation and Stability* **97**, 2262-2272 (2012).

161. Oblak P, Gonzalez-Gutierrez J, Zupančič B, Aulova A, Emri I. Processability and mechanical properties of extensively recycled high density polyethylene. *Polymer Degradation and Stability* **114**, 133-145 (2015).

162. Arena U, Mastellone ML, Perugini F. Life cycle assessment of a plastic packaging recycling system. *The International Journal of Life Cycle Assessment* **8**, 92-98 (2003).

163. Brouwer M, Picuno C, Thoden van Velzen EU, Kuchta K, De Meester S, Ragaert K. The impact of collection portfolio expansion on key performance indicators of the Dutch recycling system for Post-Consumer Plastic Packaging Waste, a comparison between 2014 and 2017. *Waste Management* **100**, 112-121 (2019).

164. Faraca G, Astrup T. Plastic waste from recycling centres: Characterisation and evaluation of plastic recyclability. *Waste Management* **95**, 388-398 (2019).

165. *Plinke E, Wenk N, Wolff G, Castiglione D, Palmark M. Mechanical Recycling of PVC Wastes: Study for DG XI of the European Commission. https://ec.europa.eu/environment/pdf/waste/studies/pvc/mech_recylce.pdf (2000).*
