# Supplementary material for: Economic viability requires higher recycling rates for imported plastic waste than expected
Source: Nat Commun. 2024 Aug 31;15:7578. doi: 10.1038/s41467-024-51923-4 (PMC11365935; doi:10.1038/s41467-024-51923-4)
Supplement: Supplementary file 3 — Reporting Summary [file 41467_2024_51923_MOESM3_ESM.pdf]

nature portfolio

Corresponding author(s): Kai Li

Last updated by author(s): Aug 5, 2024

Reporting Summary

Nature Portfolio wishes to improve the reproducibility of the work that we publish. This form provides structure for consistency and transparency in reporting. For further information on Nature Portfolio policies, see our Editorial Policies and the Editorial Policy Checklist.

Statistics

For all statistical analyses, confirm that the following items are present in the figure legend, table legend, main text, or Methods section.

n/a	Confirmed	
☐	☒	The exact sample size (*n*) for each experimental group/condition, given as a discrete number and unit of measurement
☒	☐	A statement on whether measurements were taken from distinct samples or whether the same sample was measured repeatedly
☒	☐	The statistical test(s) used AND whether they are one- or two-sided<br>*Only common tests should be described solely by name; describe more complex techniques in the Methods section.*
☒	☐	A description of all covariates tested
☐	☒	A description of any assumptions or corrections, such as tests of normality and adjustment for multiple comparisons
☐	☒	A full description of the statistical parameters including central tendency (e.g. means) or other basic estimates (e.g. regression coefficient) AND variation (e.g. standard deviation) or associated estimates of uncertainty (e.g. confidence intervals)
☒	☐	For null hypothesis testing, the test statistic (e.g. *F*, *t*, *r*) with confidence intervals, effect sizes, degrees of freedom and *P* value noted<br>*Give P values as exact values whenever suitable.*
☒	☐	For Bayesian analysis, information on the choice of priors and Markov chain Monte Carlo settings
☒	☐	For hierarchical and complex designs, identification of the appropriate level for tests and full reporting of outcomes
☒	☐	Estimates of effect sizes (e.g. Cohen's *d*, Pearson's *r*), indicating how they were calculated

Our web collection on statistics for biologists contains articles on many of the points above.

Software and code

Policy information about availability of computer code

Data collection	All the Python codes along with a catalogue have been deposited in Zenodo under the accession code DOI link https://zenodo.org/doi/10.5281/zenodo.8328894.
Data analysis	The Python code is ideally run on Python IDE e.g. Spyder with all versions.

For manuscripts utilizing custom algorithms or software that are central to the research but not yet described in published literature, software must be made available to editors and reviewers. We strongly encourage code deposition in a community repository (e.g. GitHub). See the Nature Portfolio guidelines for submitting code & software for further information.

Data

Policy information about availability of data

All manuscripts must include a data availability statement. This statement should provide the following information, where applicable:

- Accession codes, unique identifiers, or web links for publicly available datasets
- A description of any restrictions on data availability
- For clinical datasets or third party data, please ensure that the statement adheres to our policy

The data that supports the findings of this study are provided in the main text and supplementary information files. All the data has been deposited in Zenodo under the accession code DOI link https://zenodo.org/doi/10.5281/zenodo.8328894. Source data are provided with this paper.

Research involving human participants, their data, or biological material

Policy information about studies with human participants or human data. See also policy information about sex, gender (identity/presentation), and sexual orientation and race, ethnicity and racism.

Reporting on sex and gender	N/A
Reporting on race, ethnicity, or other socially relevant groupings	N/A
Population characteristics	N/A
Recruitment	N/A
Ethics oversight	N/A

Note that full information on the approval of the study protocol must also be provided in the manuscript.

Field-specific reporting

Please select the one below that is the best fit for your research. If you are not sure, read the appropriate sections before making your selection.

☐ Life sciences ☐ Behavioural & social sciences ☒ Ecological, evolutionary & environmental sciences

For a reference copy of the document with all sections, see nature.com/documents/nr-reporting-summary-flat.pdf

Ecological, evolutionary & environmental sciences study design

All studies must disclose on these points even when the disclosure is negative.

Study description	We estimate the the minimum recycling rate of imported plastic waste for the largest 22 plastic waste-importing countries from 2013-2022 using the UN Comtrade database.
Research sample	Accessing 186,861 bilateral trade records for four plastic waste types (PE, PS, PVC, and others) and six primary plastics (HDPE, LDPE, PS, PVC, PET, and PP) to derive importing cost and primary plastic values. In addition, we consider the other main plastic recycling costs including labour, electricity, and rental costs for real estate.
Sampling strategy	We require trade values for a country's imported plastic waste and its exported primary plastics, derived from the UN Comtrade database. There are two options: using trade values reported by the research country, including plastic waste import (CIF basis) and primary plastics export (FOB basis), or using mirror trade values reported by the trading partner of the research country, including plastic waste export (FOB basis) and primary plastic import (CIF basis). In calculating the RRR via the cost-benefit equation, we aim to incorporate the international transport cost for importing plastic waste while excluding the transport revenue for recycled primary plastics. Therefore, we prioritize using plastic waste imports (CIF basis) and primary plastics exports (FOB basis), both reported by the research countries. However, for a robustness check, we also include results based on mirror trade data reported by the trading partners of the 22 research countries.
Data collection	UN Comtrade database
Timing and spatial scale	2013-2022
Data exclusions	We detected trade value outliers through a distributional analysis of the value-to-mass ratio for all trade entries of specific plastic types each year. Since most of these unit price distributions follow a lognormal pattern, we transformed them into normal distributions by taking the natural logarithm (ln($/kg)). By identifying outliers greater than three standard deviations from the mean value, approximately 2.4% and 2.6% of plastic waste and primary plastic trade entries were flagged as outliers, respectively.
Reproducibility	N/A
Randomization	N/A
Blinding	N/A

Did the study involve field work? ☐ Yes ☒ No

Reporting for specific materials, systems and methods

We require information from authors about some types of materials, experimental systems and methods used in many studies. Here, indicate whether each material, system or method listed is relevant to your study. If you are not sure if a list item applies to your research, read the appropriate section before selecting a response.

Materials & experimental systems

n/a	Involved in the study
☒	☐ Antibodies
☒	☐ Eukaryotic cell lines
☒	☐ Palaeontology and archaeology
☒	☐ Animals and other organisms
☒	☐ Clinical data
☒	☐ Dual use research of concern
☒	☐ Plants

Methods

n/a	Involved in the study
☒	☐ ChIP-seq
☒	☐ Flow cytometry
☒	☐ MRI-based neuroimaging

Plants

Seed stocks	N/A
Novel plant genotypes	N/A
Authentication	N/A
